# Supplementary material for: PTGS is dispensable for the initiation of epigenetic silencing of an active transposon in Arabidopsis
Source: EMBO Rep. 2024 Nov 7;25(12):28. doi: 10.1038/s44319-024-00304-5 (PMC11624286; doi:10.1038/s44319-024-00304-5)
Supplement: Supplementary file 2 — Source data Fig. 1 [file 44319_2024_304_MOESM2_ESM.zip › Figure 1/1E/Northern blots 1E.pdf]

Same membrane was re-probed multiple times against indicated targets, cropped areas for inclusion in the final figure are indicated in red squares. (lane/sample description at the end of the document)

@GAG

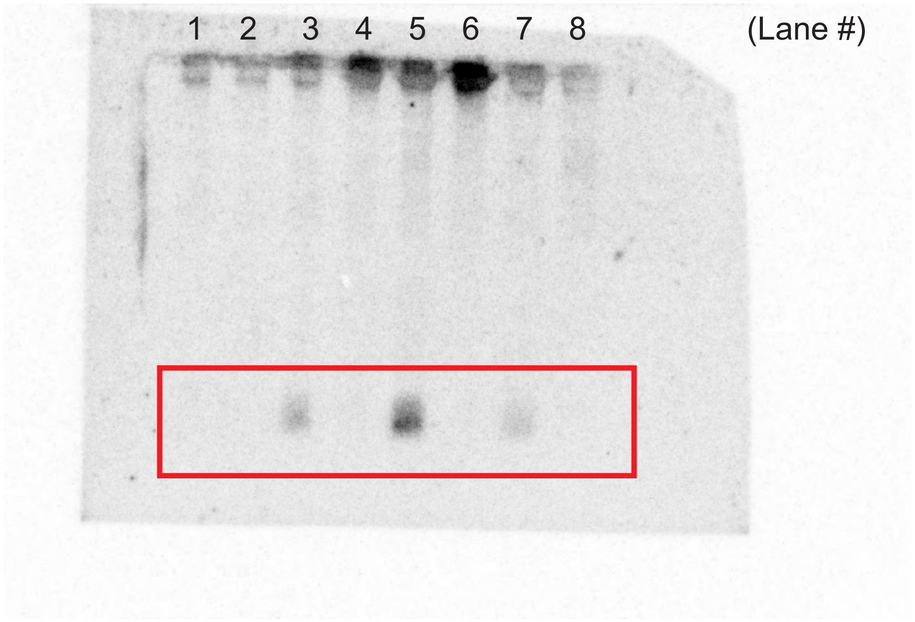

@LTR

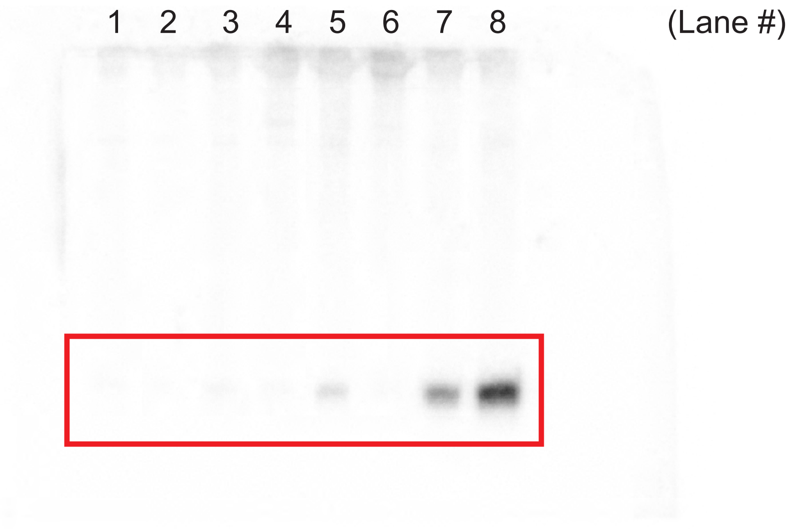

@tasiRNA255

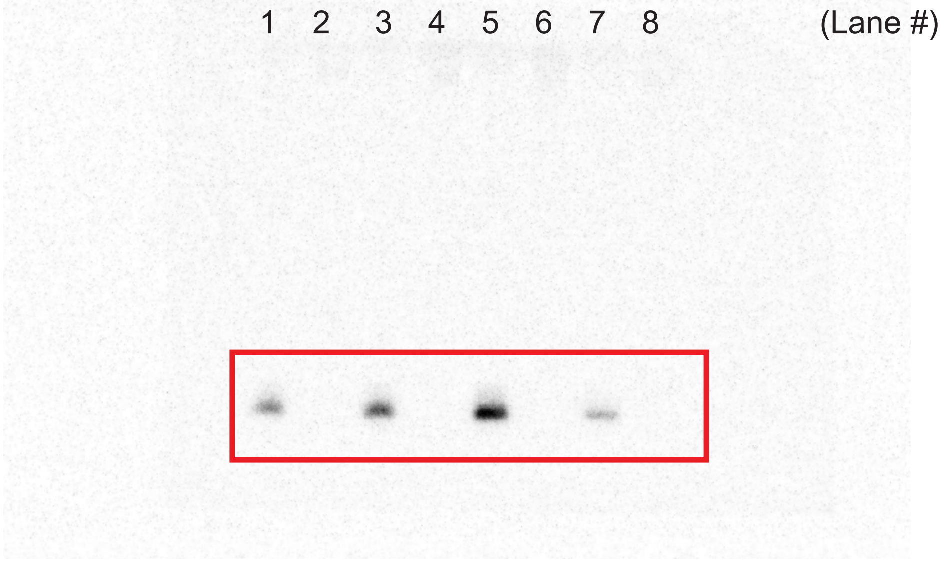

@U6 + miRNA171 (short exposure)

Shown as U6

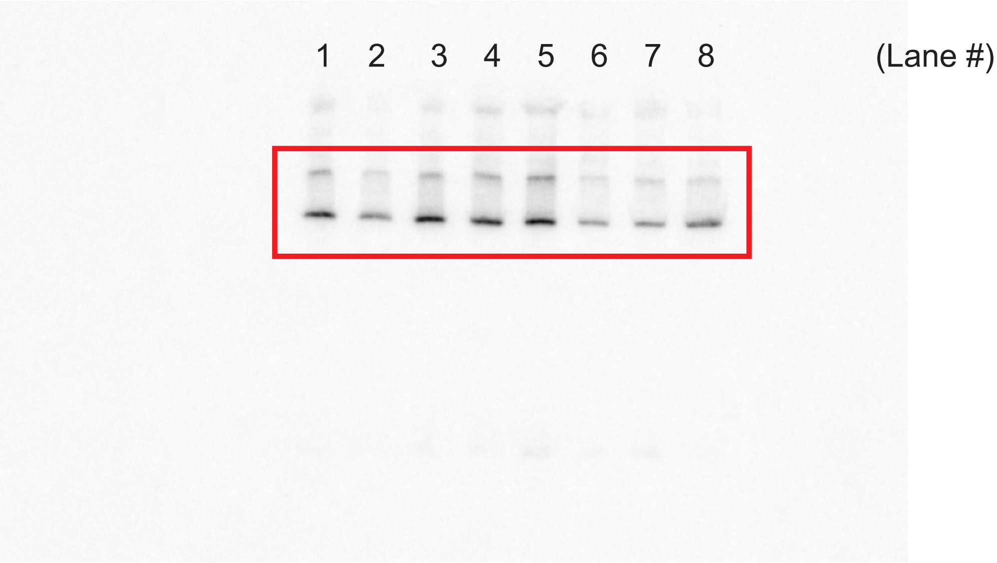

@U6 + miRNA171 (long exposure)

Shown as miR171

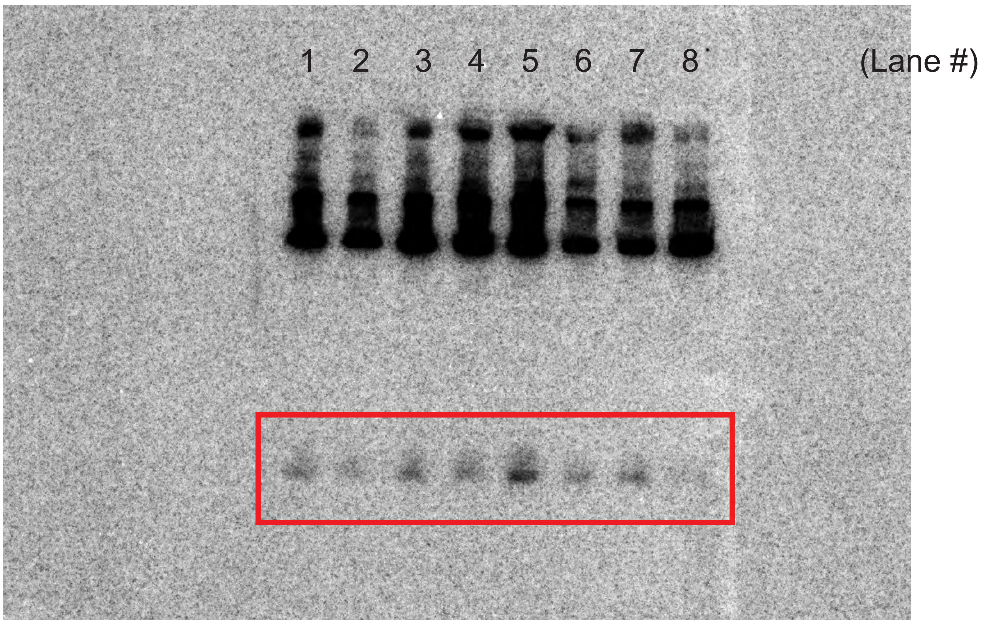

Lane sample loading:

rdr6 x epi15 for bulks of mutant and WT plants at generations F2, F4 and F6

1) Col-0
2) *rdr6*
3) F2 *RDR6*
4) F2 *rdr6*
5) F4 *RDR6*
6) F4 *rdr6*
7) F6 *RDR6*
8) F6 *rdr6*
