# Supplementary material for: PTGS is dispensable for the initiation of epigenetic silencing of an active transposon in Arabidopsis
Source: EMBO Rep. 2024 Nov 7;25(12):28. doi: 10.1038/s44319-024-00304-5 (PMC11624286; doi:10.1038/s44319-024-00304-5)
Supplement: Supplementary file 2 — Source data Fig. 1 [file 44319_2024_304_MOESM2_ESM.zip › Figure 1/1E/Raw blot images/infos_northern_raw_files.rtf]

Raw files for northerns (membrane mtM028)mtM028 -  rdr6 x epi15 F8 line, for bulks of mutant and WT plants at generations F2, F4 and F6Loading (form left to right) - Col-0, rdr6, F2+, F2-, F4+, F4-, F6+, F6-
