# Supplementary figures and images for: PTGS is dispensable for the initiation of epigenetic silencing of an active transposon in Arabidopsis
Source: EMBO Rep. 2024 Nov 7;25(12):28. doi: 10.1038/s44319-024-00304-5 (PMC11624286; doi:10.1038/s44319-024-00304-5)

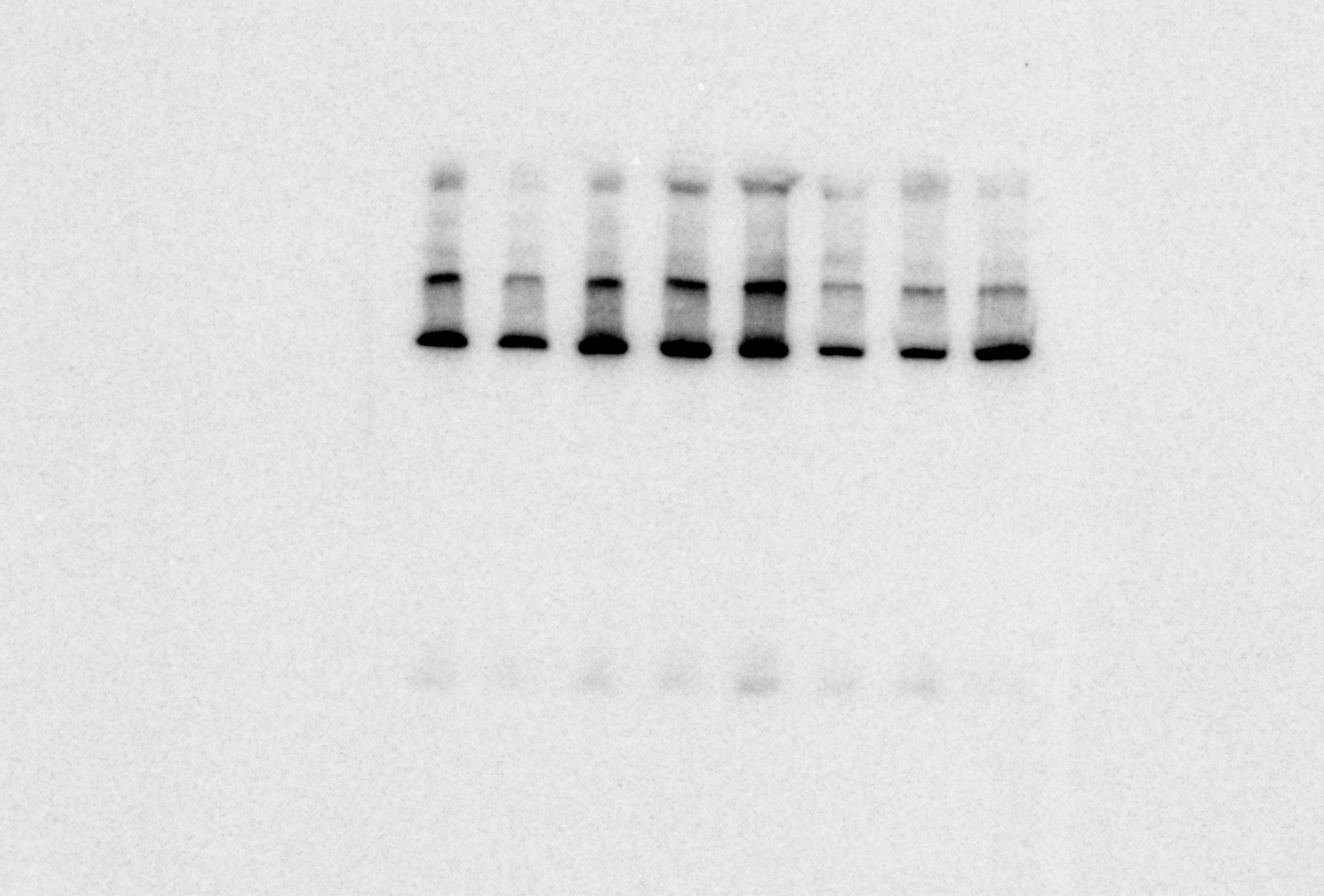

Supplement: Supplementary file 2 — Source data Fig. 1 [file 44319_2024_304_MOESM2_ESM.zip › Figure 1/1E/Raw blot images/RDR6-rdr6-EVD gens_@miR171+U6_061222.tif]

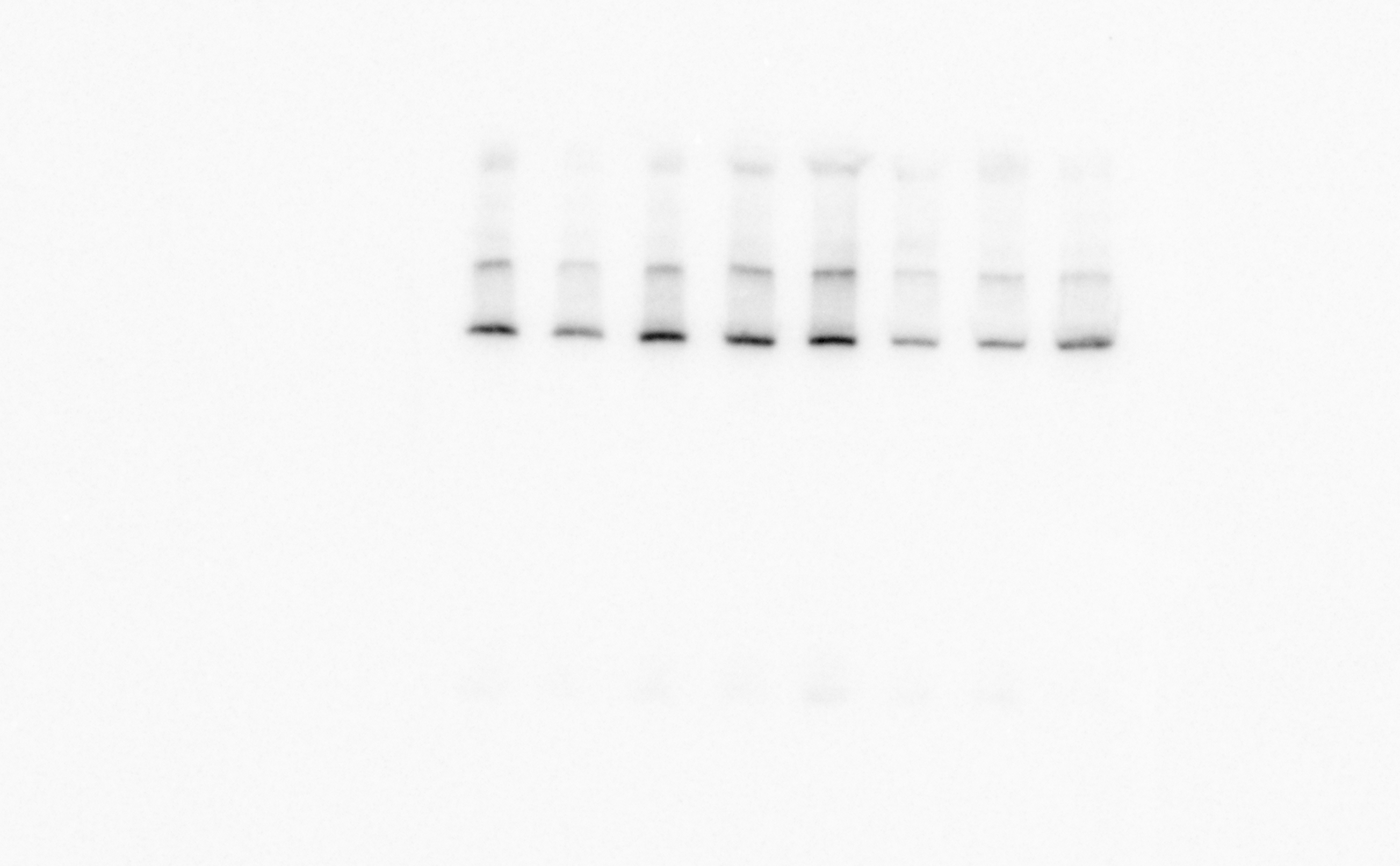

Supplement: Supplementary file 2 — Source data Fig. 1 [file 44319_2024_304_MOESM2_ESM.zip › Figure 1/1E/Raw blot images/RDR6-rdr6-EVD gens_@miR171+U6_061222 short exp.tif]

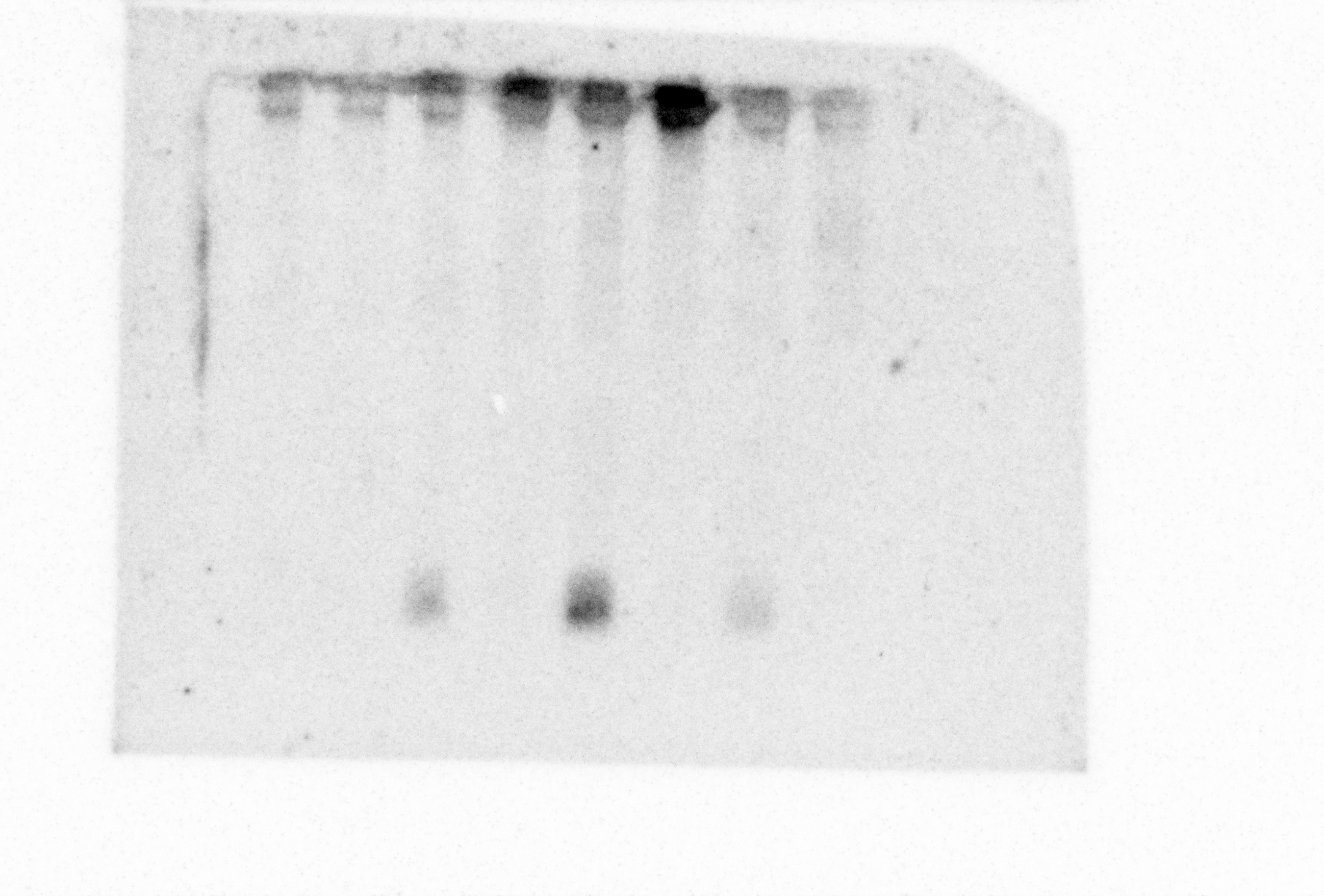

Supplement: Supplementary file 2 — Source data Fig. 1 [file 44319_2024_304_MOESM2_ESM.zip › Figure 1/1E/Raw blot images/RDR6-rdr6-EVD gens_@EVD-GAG_170921.tif]

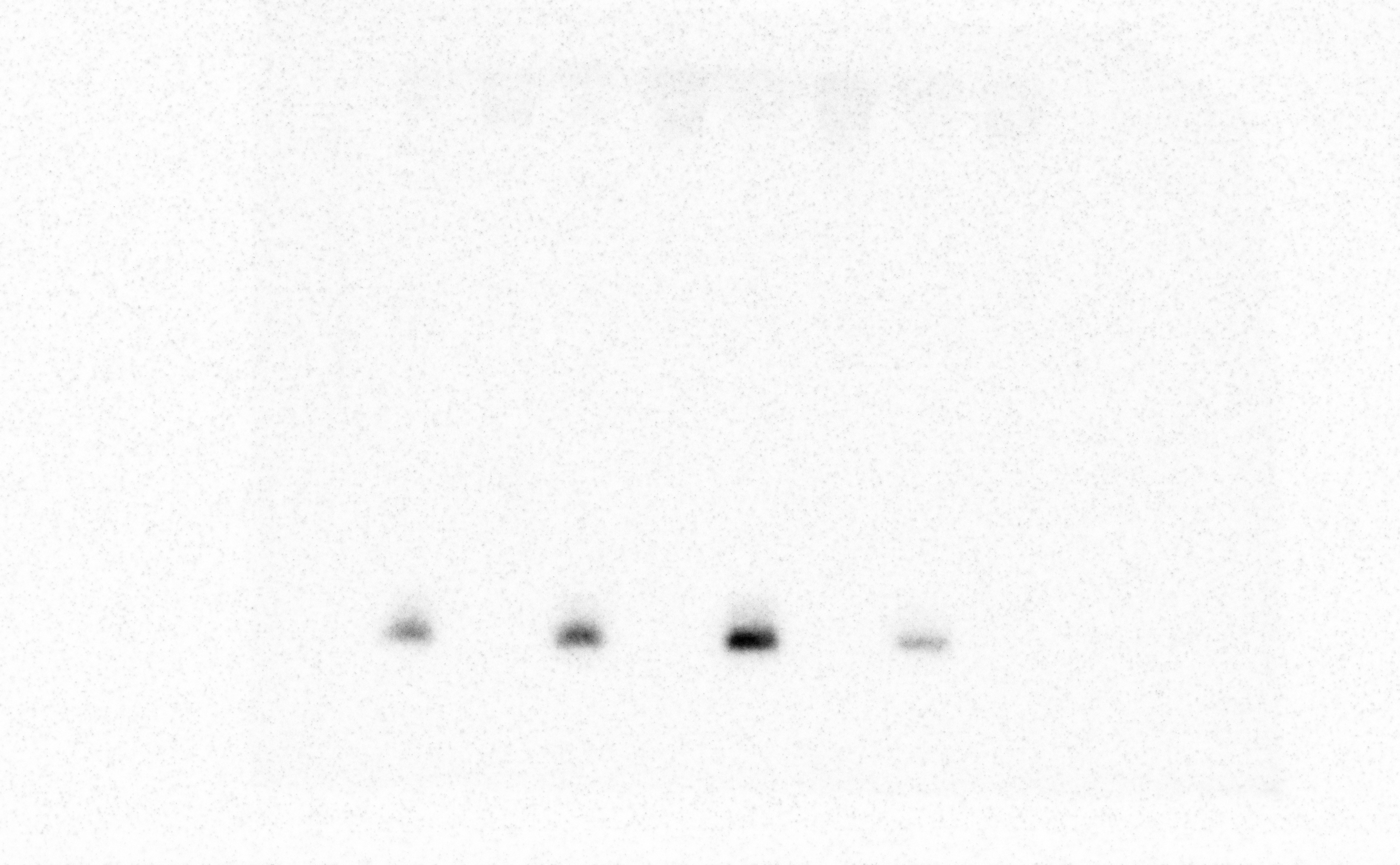

Supplement: Supplementary file 2 — Source data Fig. 1 [file 44319_2024_304_MOESM2_ESM.zip › Figure 1/1E/Raw blot images/RDR6-rdr6-EVD gens_@tasi255_ON_240921.tif]

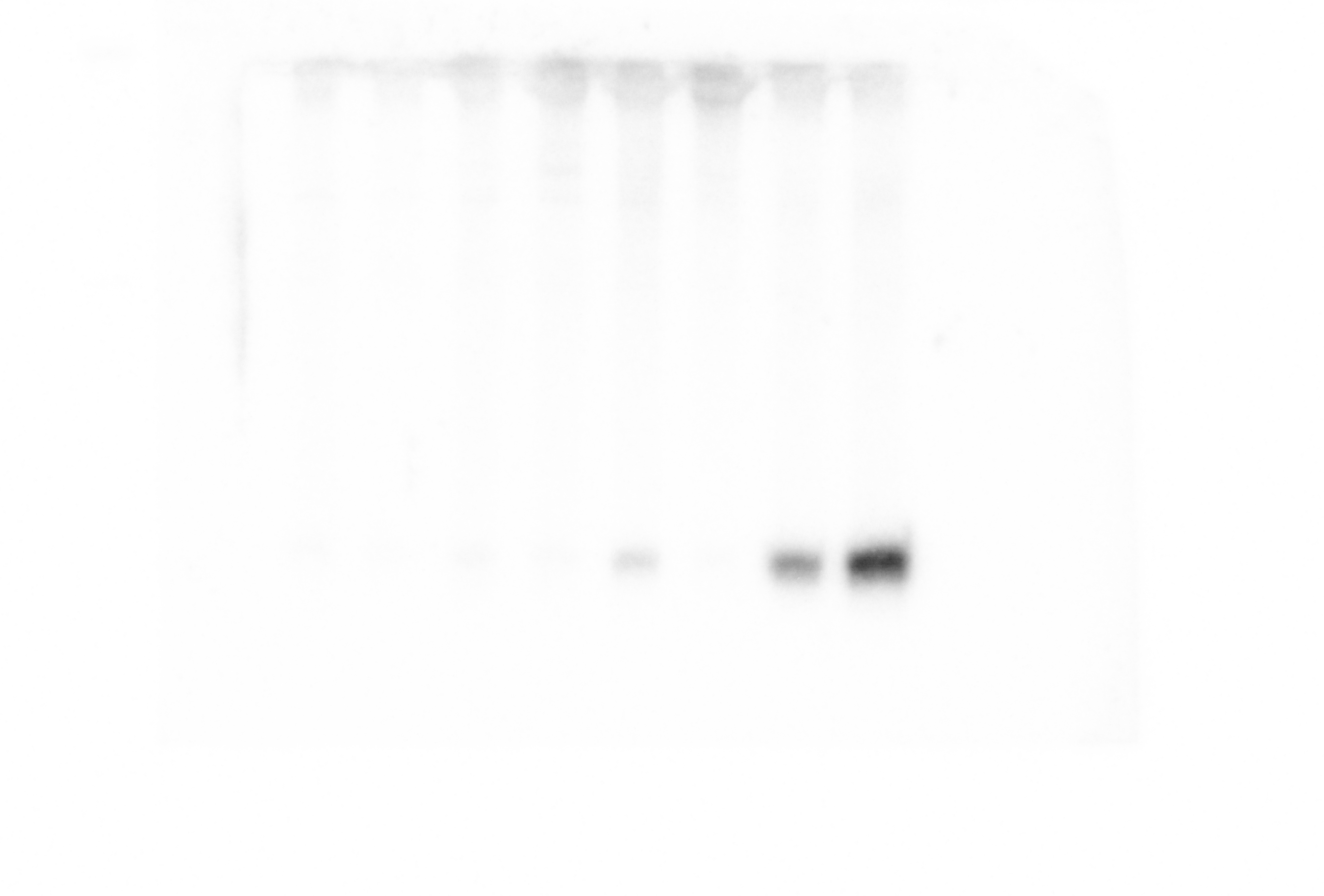

Supplement: Supplementary file 2 — Source data Fig. 1 [file 44319_2024_304_MOESM2_ESM.zip › Figure 1/1E/Raw blot images/RDR6-rdr6-EVD gens_@EVD-LTR_ON_150921.tif]

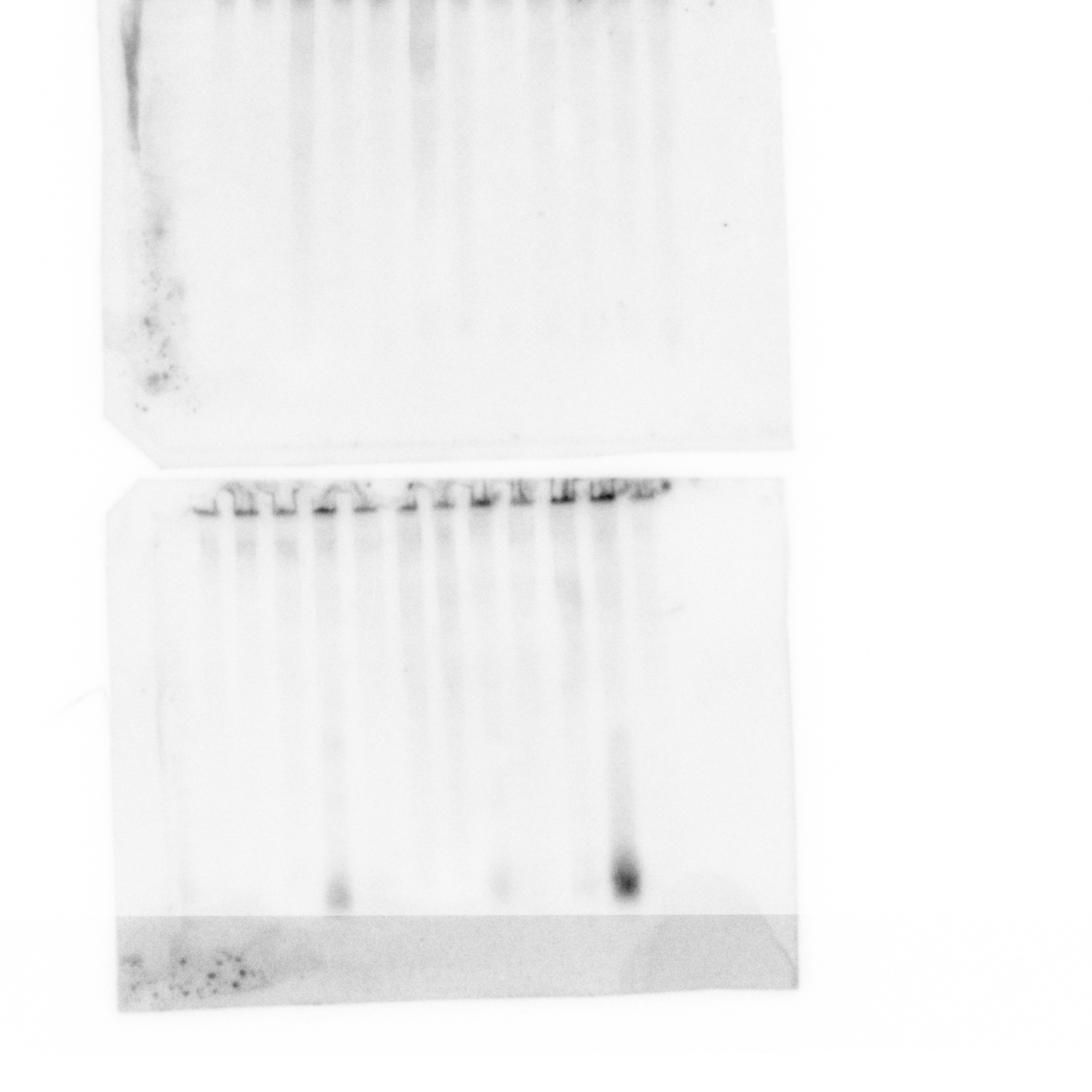

Supplement: Supplementary file 3 — Source data Fig. 2 [file 44319_2024_304_MOESM3_ESM.zip › Figure 2/2A/Raw blot images 2A/20220217_RDR6-rdr6-EVD_F6_indv @ GAG_scann2.tif]

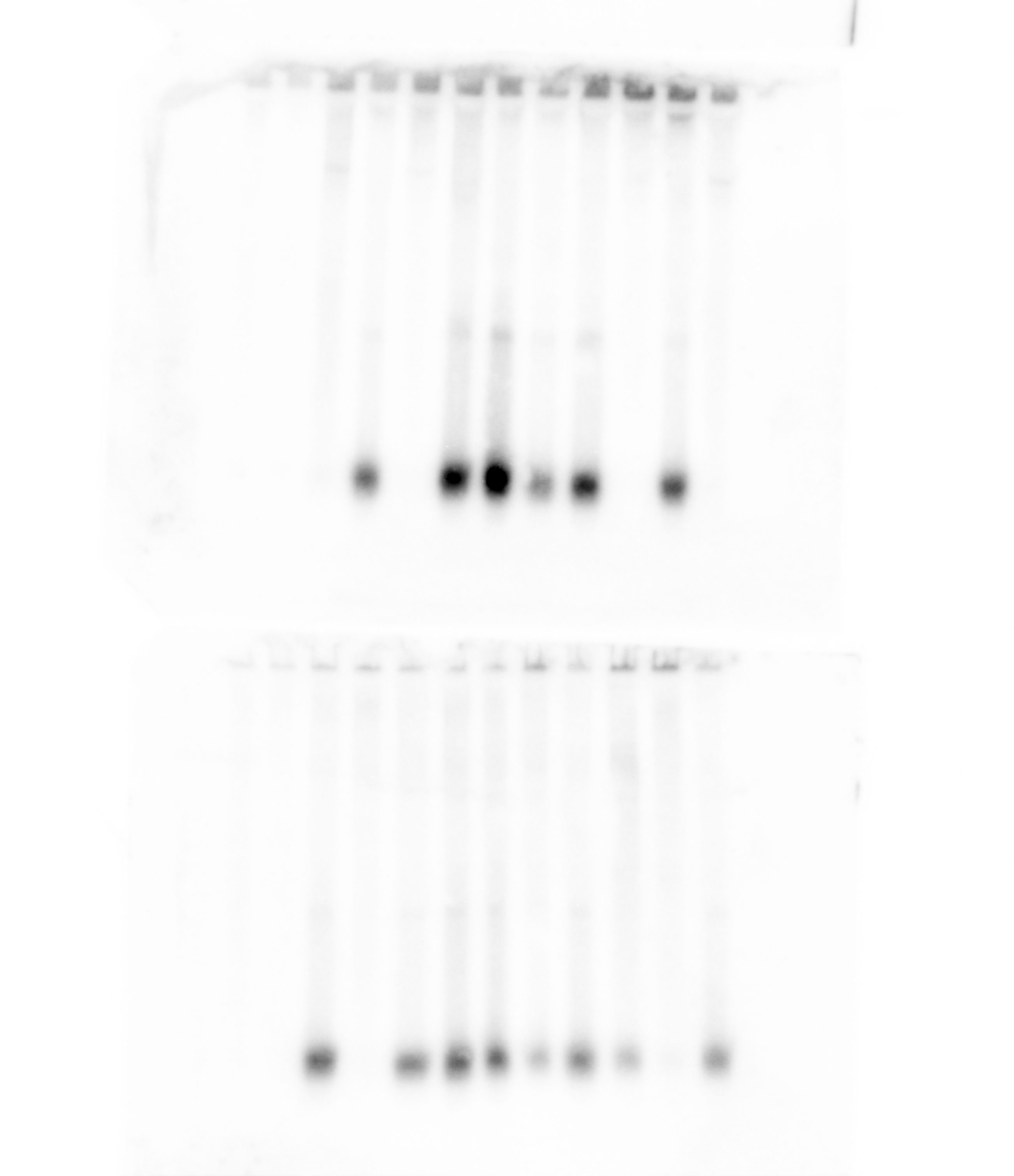

Supplement: Supplementary file 3 — Source data Fig. 2 [file 44319_2024_304_MOESM3_ESM.zip › Figure 2/2A/Raw blot images 2A/20220217_RDR6-rdr6-EVD_F6_indv @ LTR.tif]

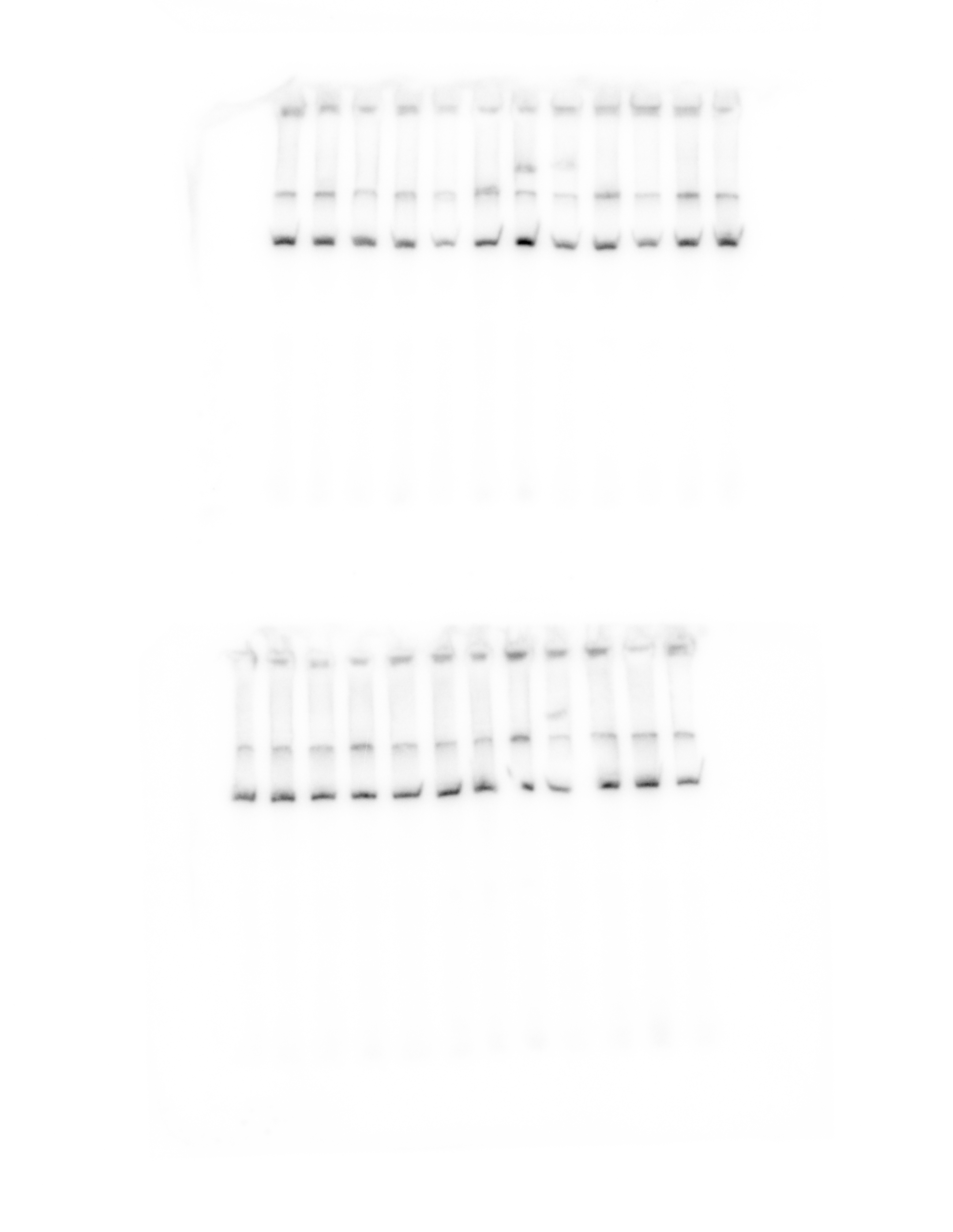

Supplement: Supplementary file 3 — Source data Fig. 2 [file 44319_2024_304_MOESM3_ESM.zip › Figure 2/2A/Raw blot images 2A/20220217_RDR6-rdr6-EVD_F6_indv @ U6+miR171.tif]

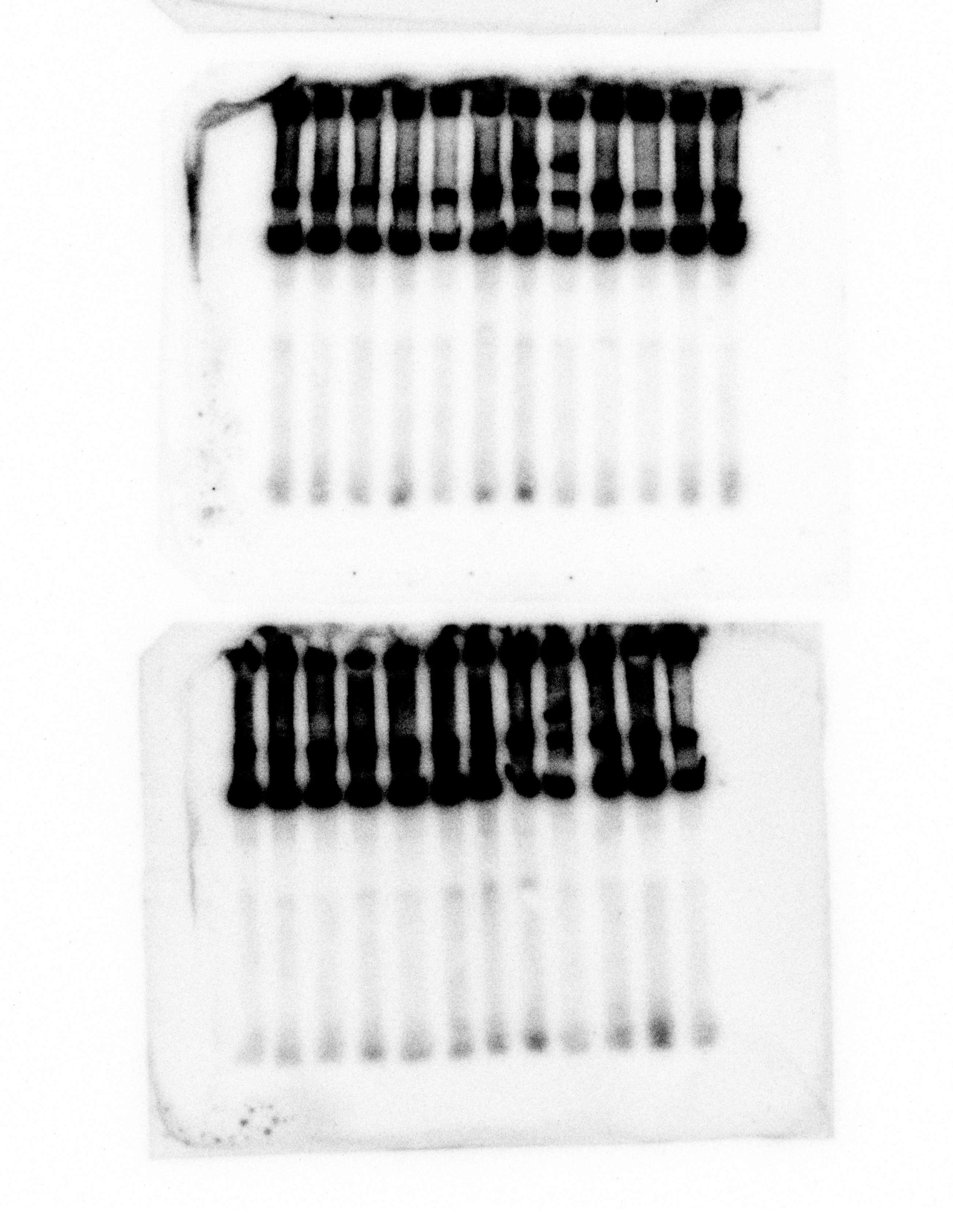

Supplement: Supplementary file 3 — Source data Fig. 2 [file 44319_2024_304_MOESM3_ESM.zip › Figure 2/2A/Raw blot images 2A/20220217_RDR6-rdr6-EVD_F6_indv @ U6+miR171_high-exp.tif]

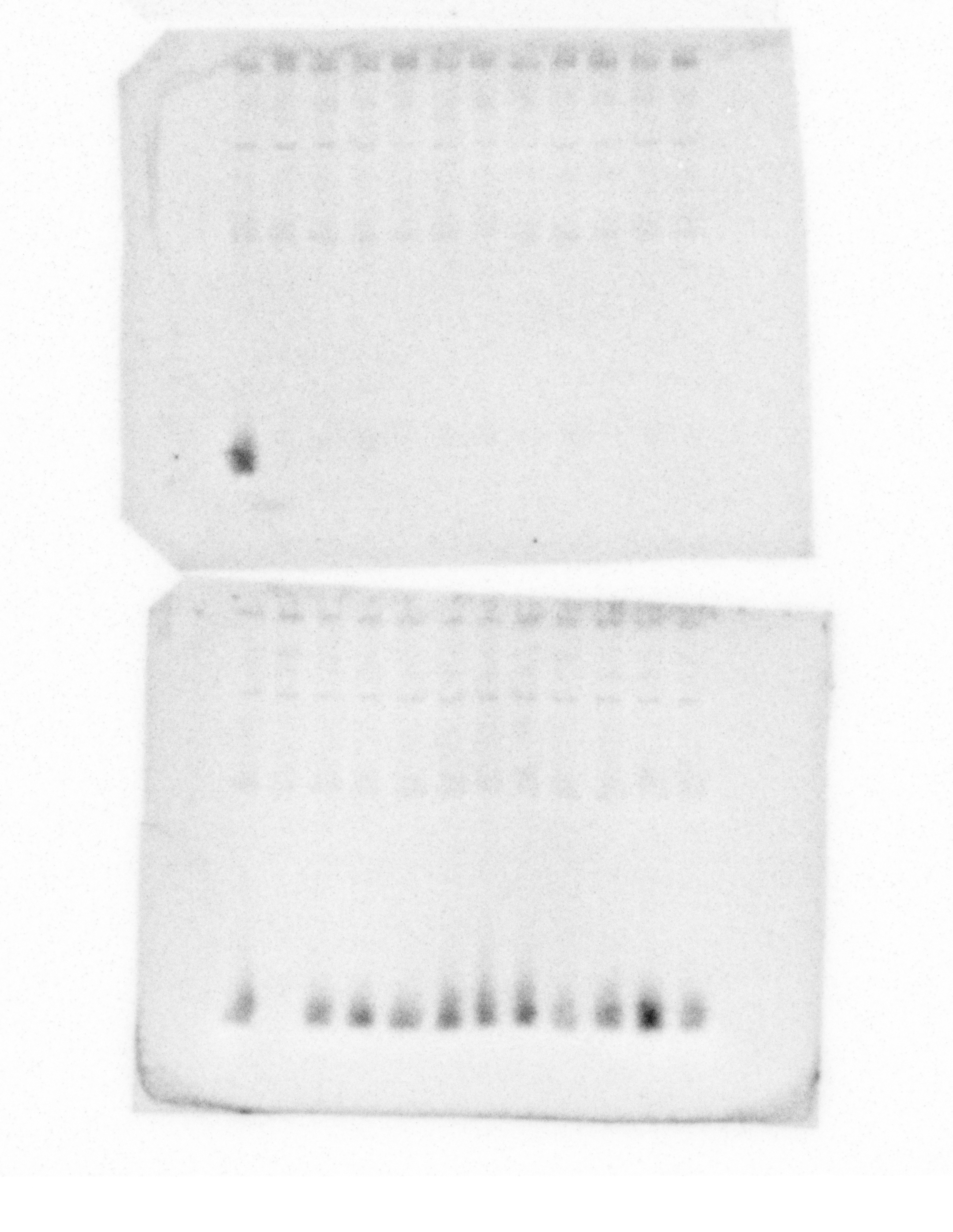

Supplement: Supplementary file 3 — Source data Fig. 2 [file 44319_2024_304_MOESM3_ESM.zip › Figure 2/2A/Raw blot images 2A/20220217_RDR6-rdr6-EVD_F6_indv @ tasiR255.tif]

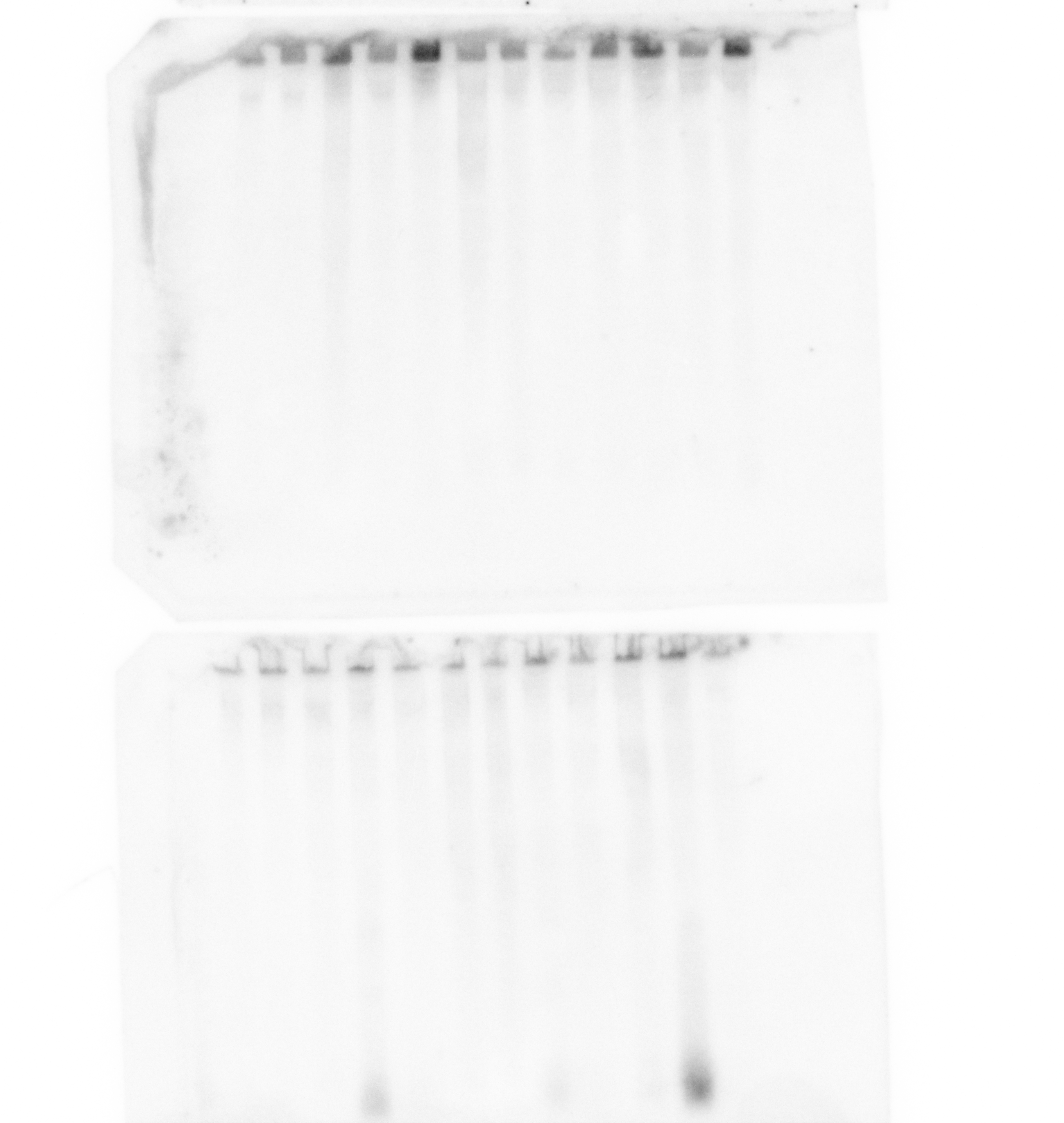

Supplement: Supplementary file 3 — Source data Fig. 2 [file 44319_2024_304_MOESM3_ESM.zip › Figure 2/2A/Raw blot images 2A/20220217_RDR6-rdr6-EVD_F6_indv @ GAG_scan1.tif]

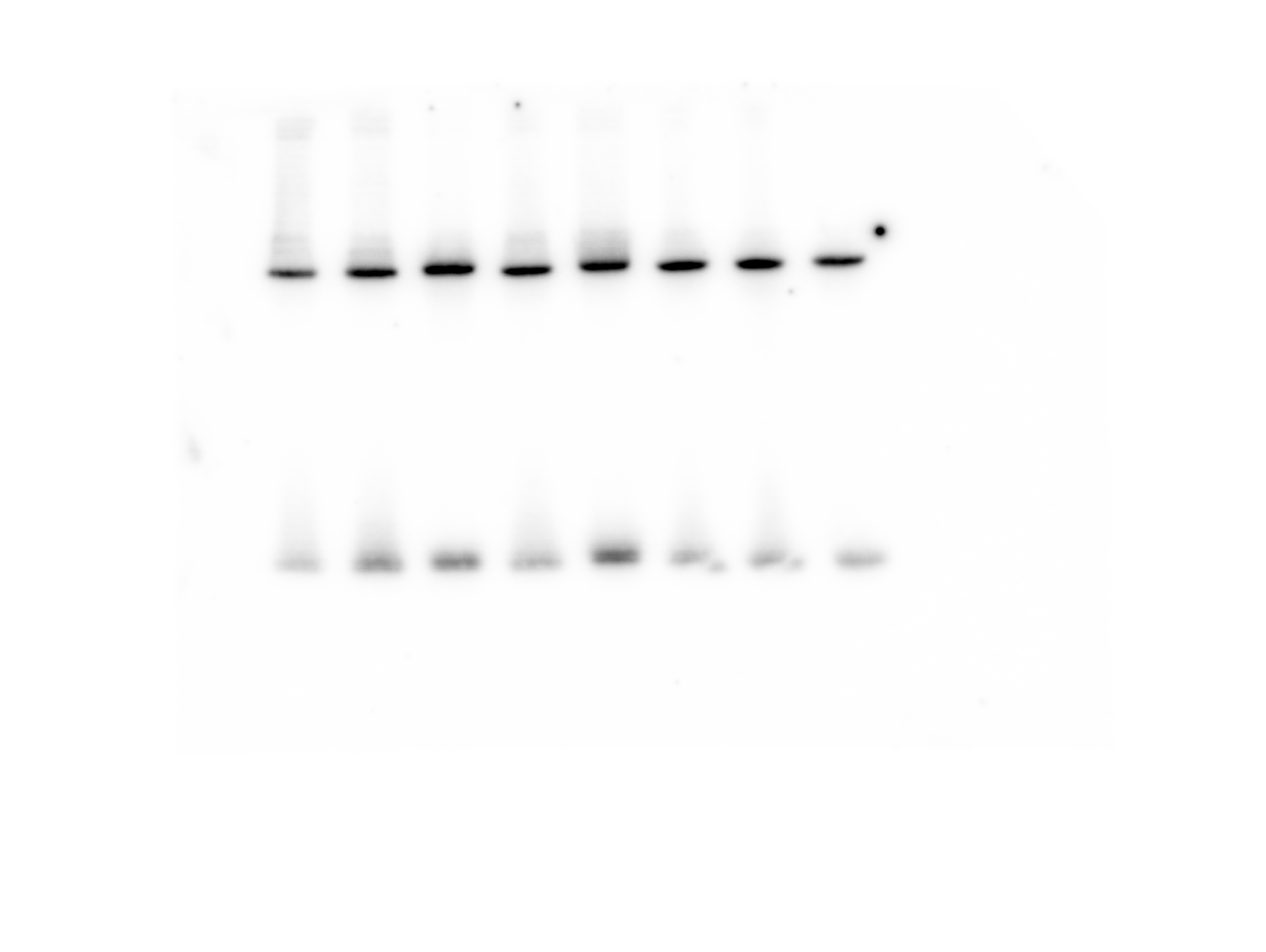

Supplement: Supplementary file 7 — Source data Fig. 6 [file 44319_2024_304_MOESM7_ESM.zip › Figure 6/6D/Raw blot images 6D/MT008_NRPE-nrpe-EVD F6 @ U6+mir171.tif]

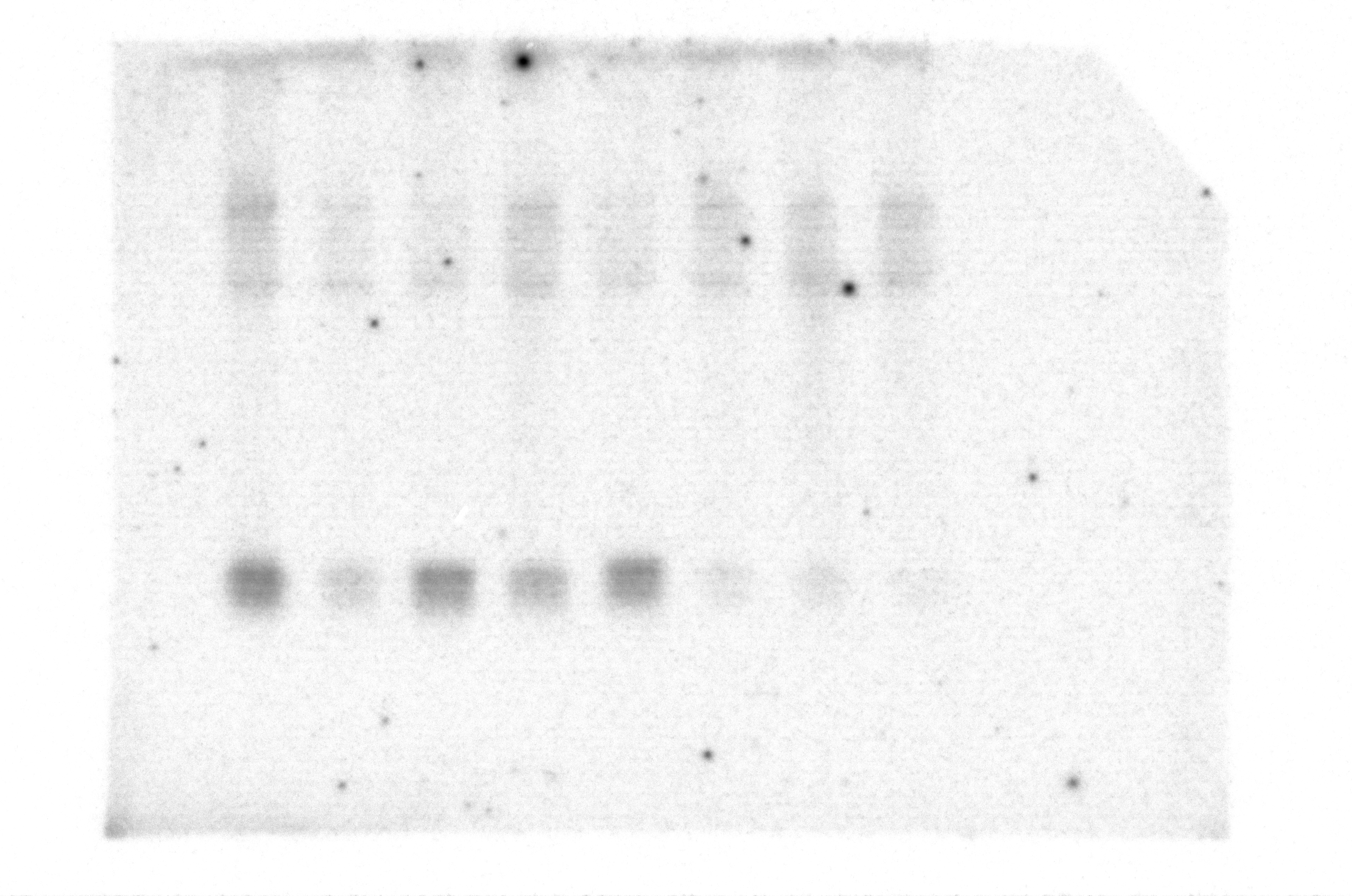

Supplement: Supplementary file 7 — Source data Fig. 6 [file 44319_2024_304_MOESM7_ESM.zip › Figure 6/6D/Raw blot images 6D/MT008_NRPE-nrpe-EVD F6 @ siR1003.tif]

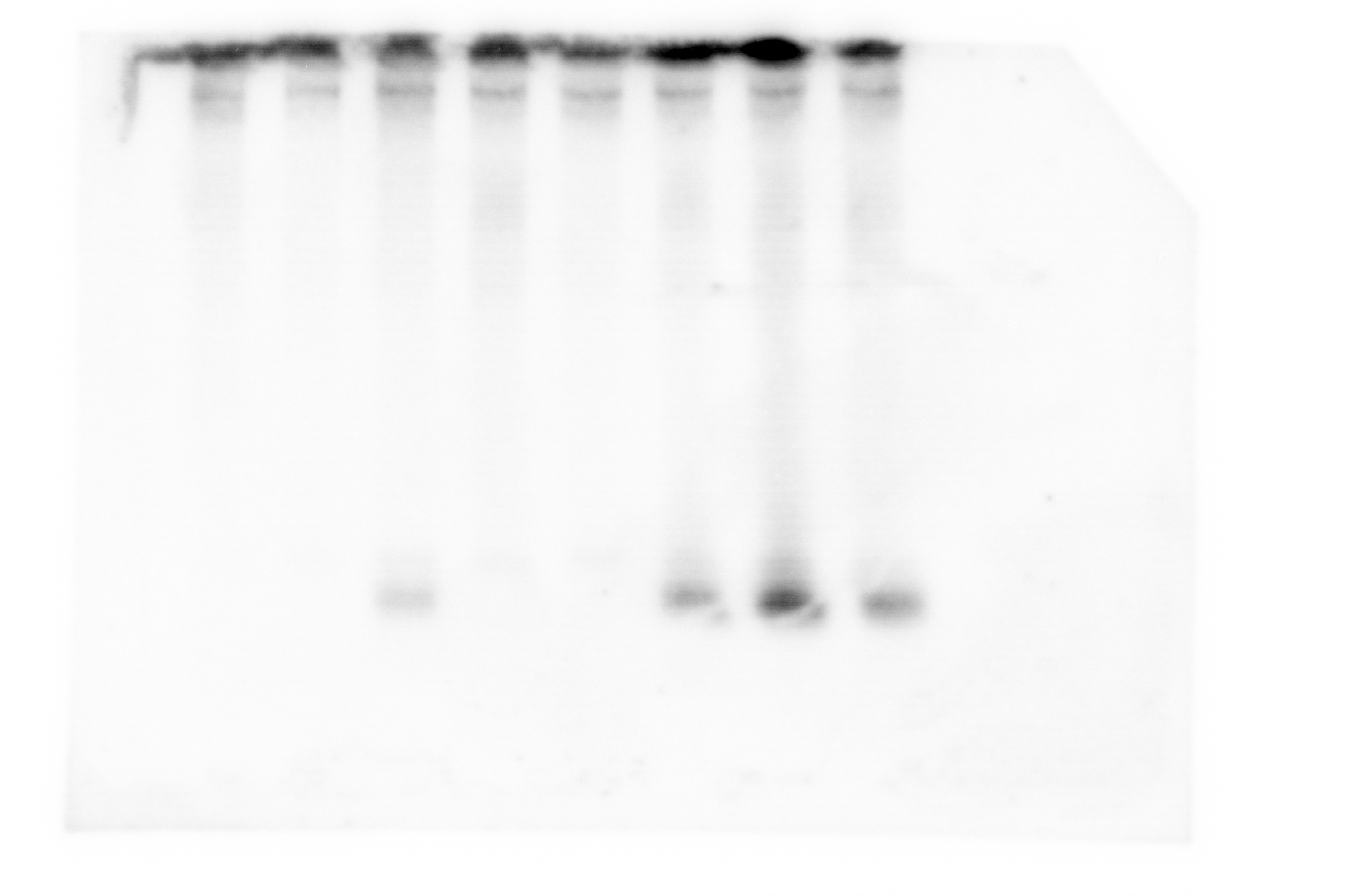

Supplement: Supplementary file 7 — Source data Fig. 6 [file 44319_2024_304_MOESM7_ESM.zip › Figure 6/6D/Raw blot images 6D/MT008_NRPE-nrpe-EVD F6 @ EVD-GAG.tif]

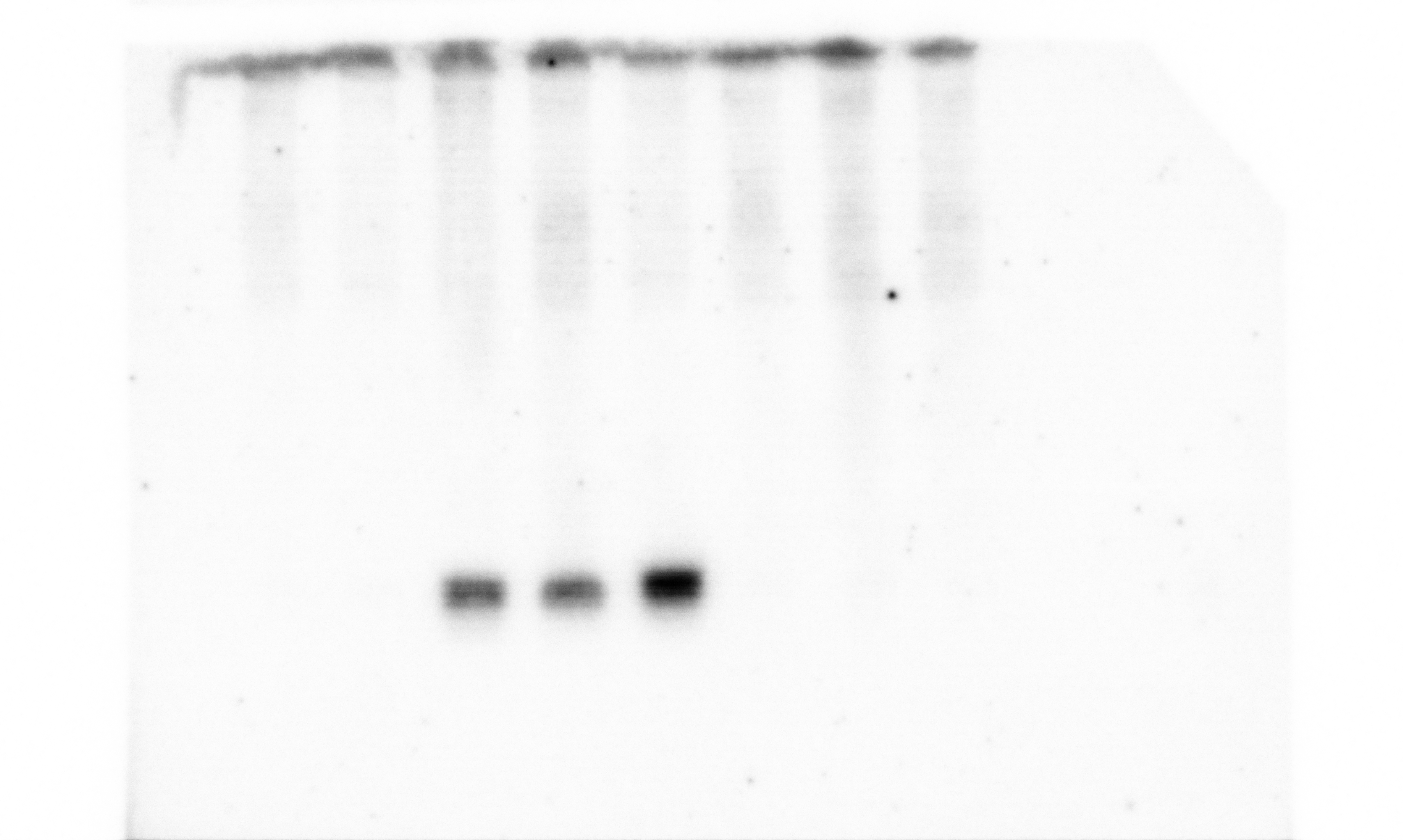

Supplement: Supplementary file 7 — Source data Fig. 6 [file 44319_2024_304_MOESM7_ESM.zip › Figure 6/6D/Raw blot images 6D/MT008_NRPE-nrpe-EVD F6 @ EVD-LTR.tif]

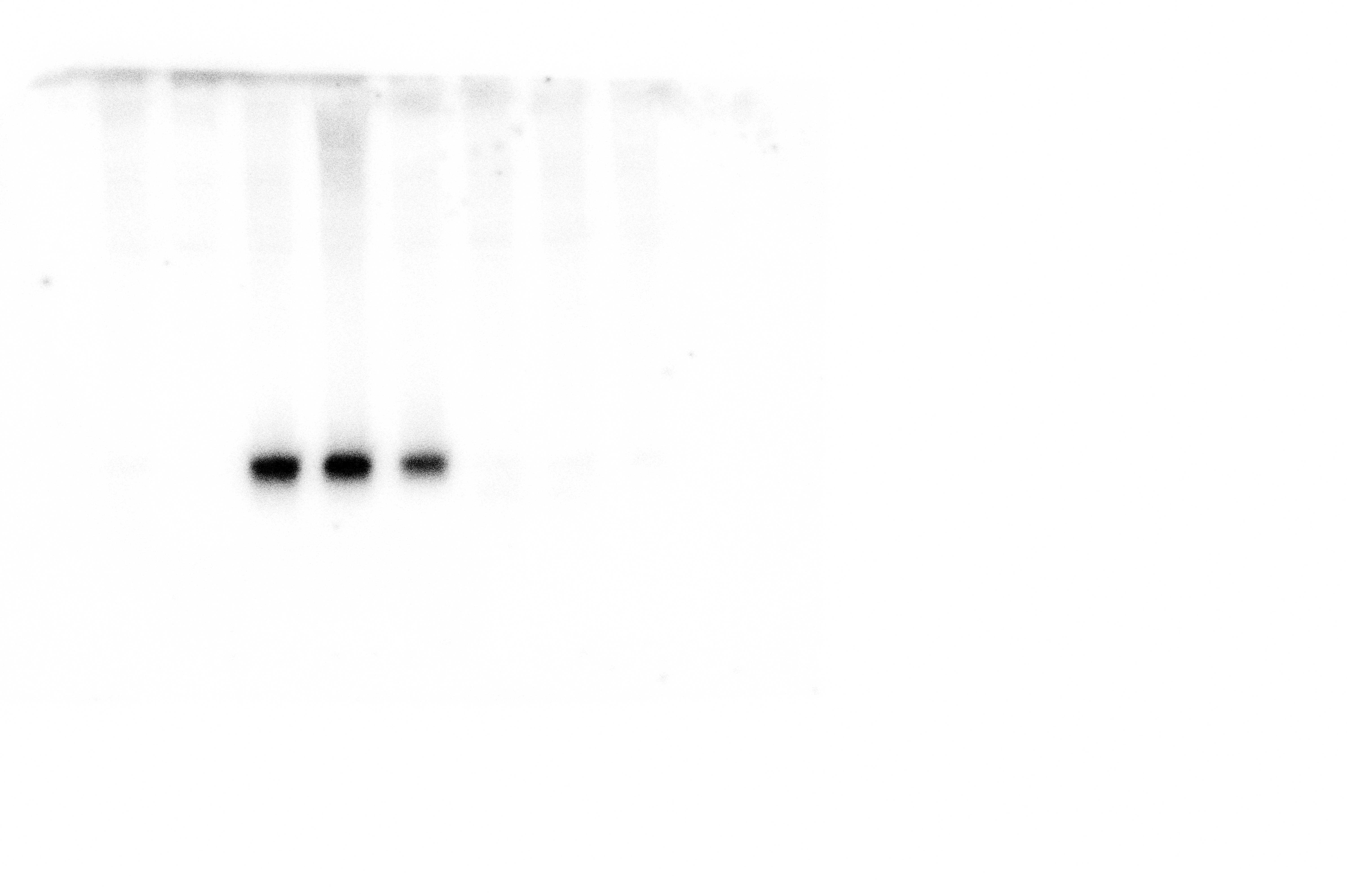

Supplement: Supplementary file 7 — Source data Fig. 6 [file 44319_2024_304_MOESM7_ESM.zip › Figure 6/6C/Raw blot images 6C/MT002_NRPD-nrpd-EVD F6 @ EVD-LTR.tif]

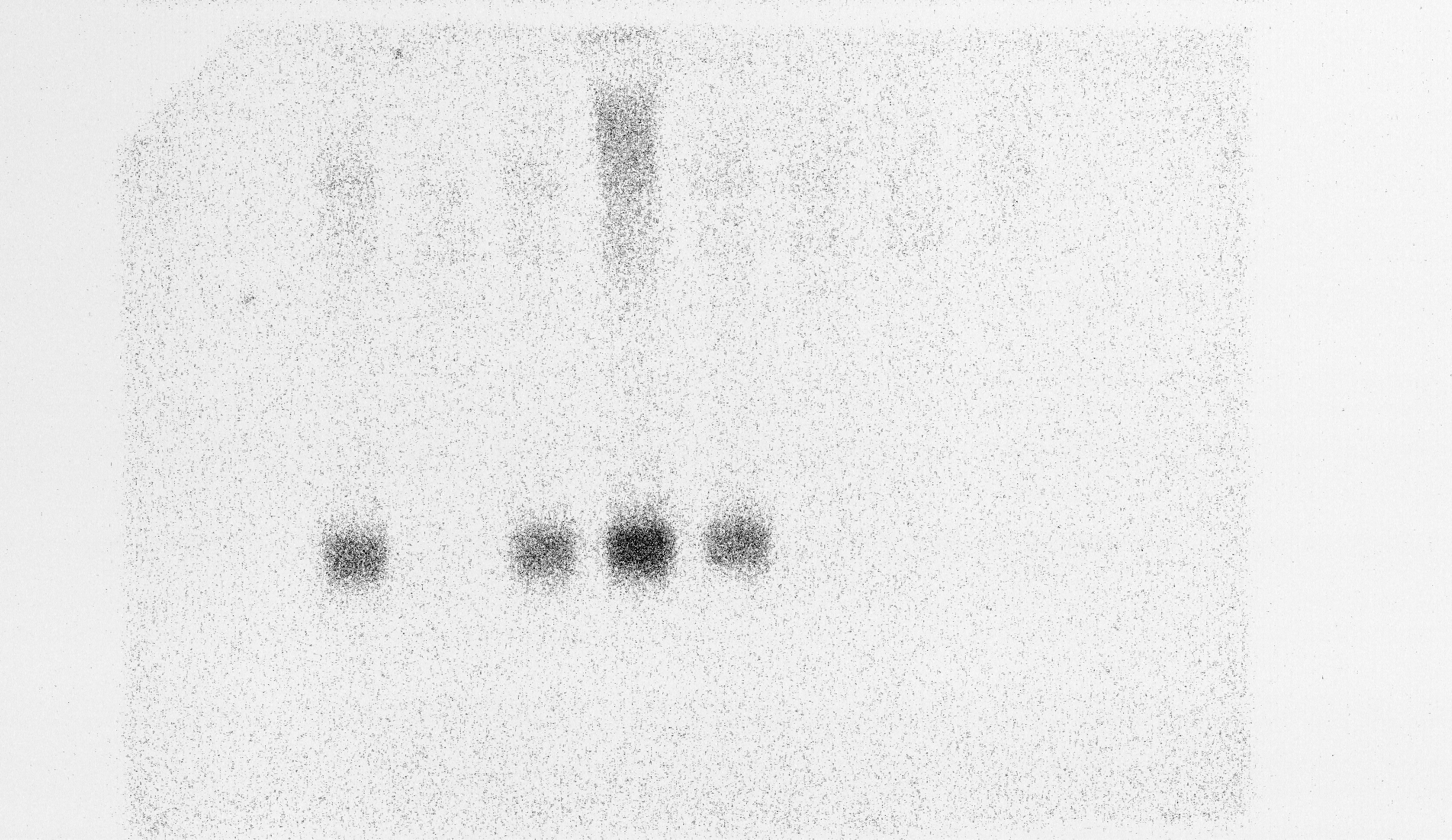

Supplement: Supplementary file 7 — Source data Fig. 6 [file 44319_2024_304_MOESM7_ESM.zip › Figure 6/6C/Raw blot images 6C/MT002_NRPD-nrpd-EVD F6 @ siR1003.tif]

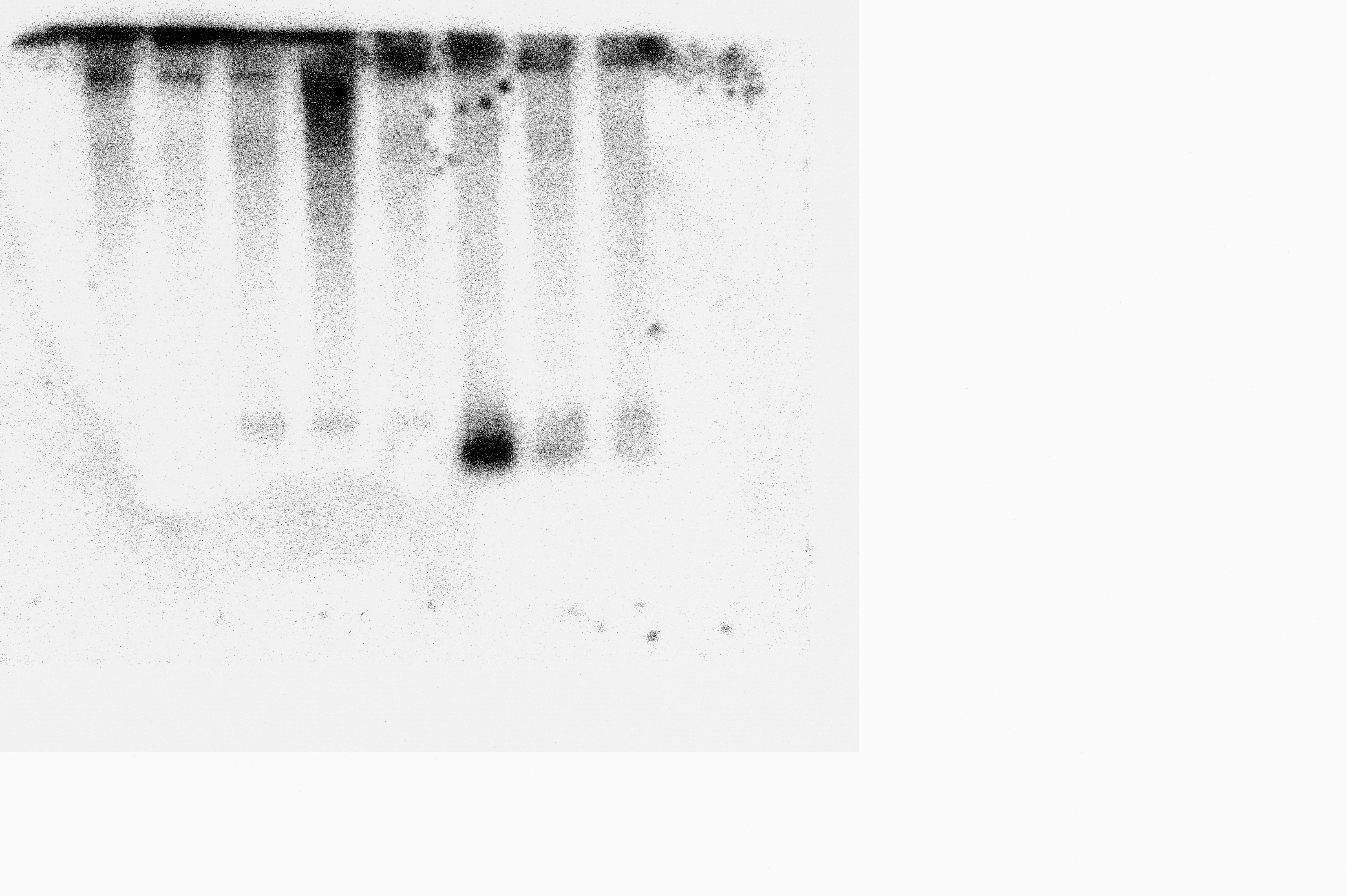

Supplement: Supplementary file 7 — Source data Fig. 6 [file 44319_2024_304_MOESM7_ESM.zip › Figure 6/6C/Raw blot images 6C/MT002_NRPD-nrpd-EVD F6 @ EVD-GAG.tif]

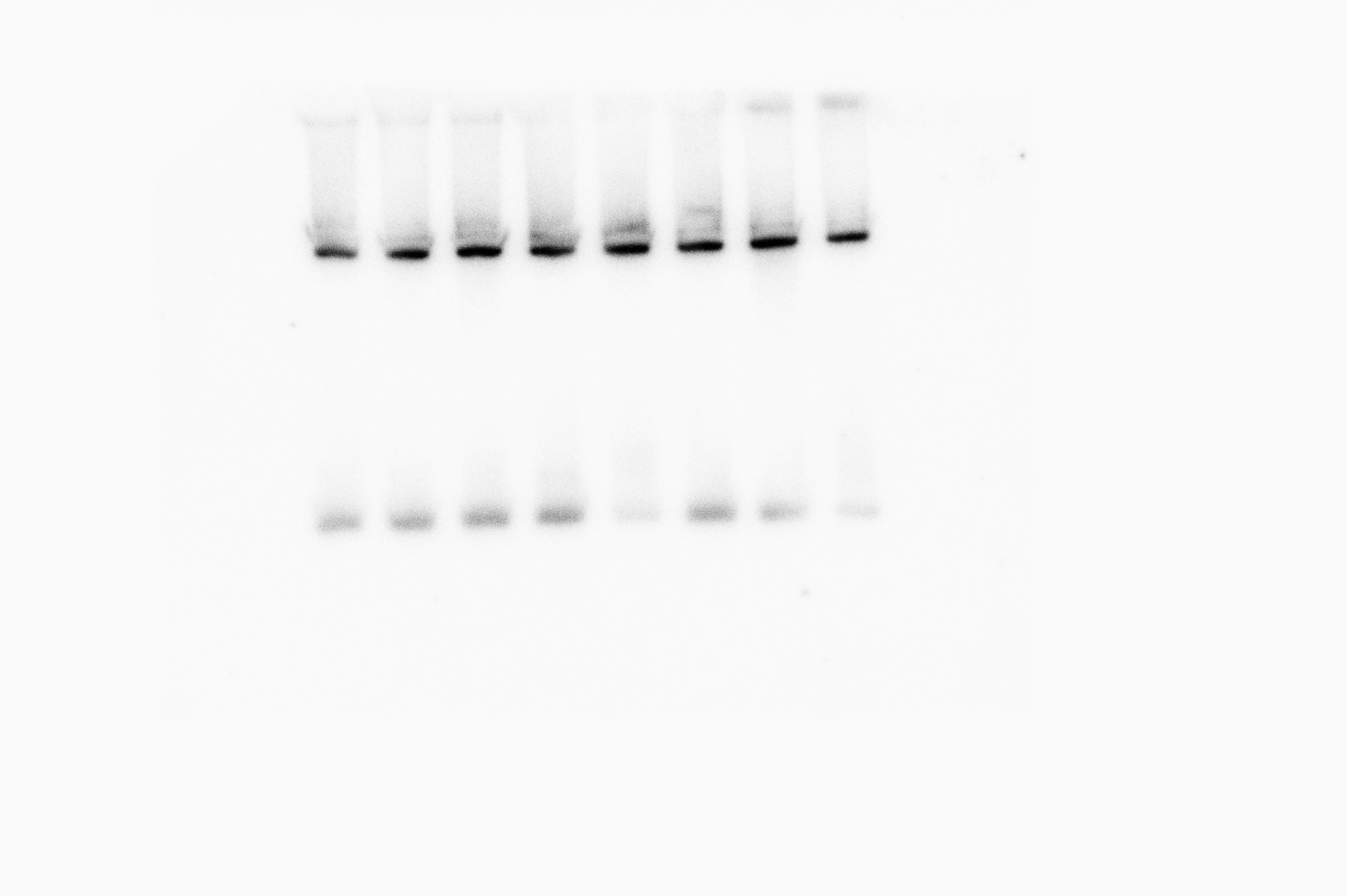

Supplement: Supplementary file 7 — Source data Fig. 6 [file 44319_2024_304_MOESM7_ESM.zip › Figure 6/6C/Raw blot images 6C/MT002_NRPD-nrpd-EVD F6 @ U6+miR171.tif]

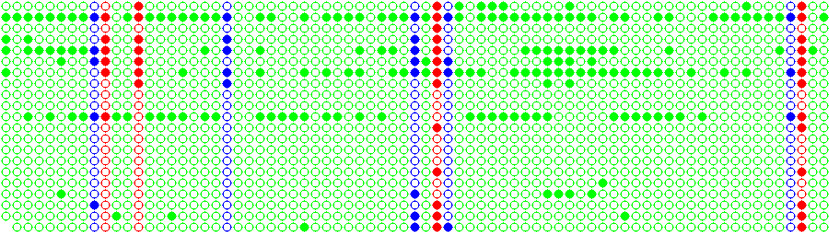

Supplement: Supplementary file 9 — Figure EV1-4 Source Data [file 44319_2024_304_MOESM9_ESM.zip › EV Figures/Figure EV4/EV4G/LTR_p5_F6+.png]

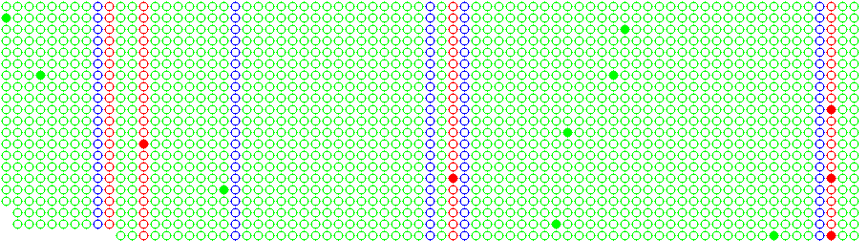

Supplement: Supplementary file 9 — Figure EV1-4 Source Data [file 44319_2024_304_MOESM9_ESM.zip › EV Figures/Figure EV4/EV4G/LTR_p5_F6-.png]

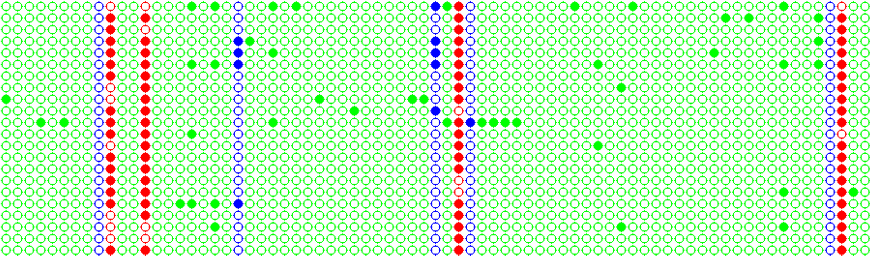

Supplement: Supplementary file 9 — Figure EV1-4 Source Data [file 44319_2024_304_MOESM9_ESM.zip › EV Figures/Figure EV4/EV4G/LTR_p5_Col-0.png]

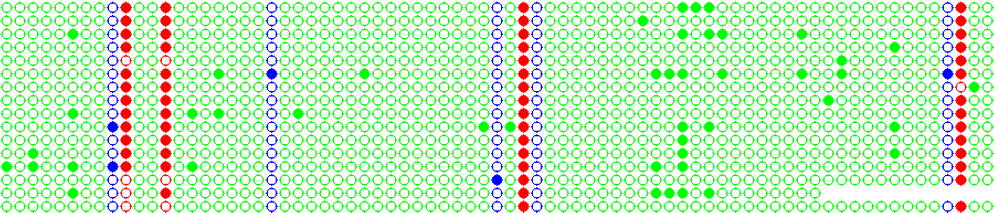

Supplement: Supplementary file 9 — Figure EV1-4 Source Data [file 44319_2024_304_MOESM9_ESM.zip › EV Figures/Figure EV4/EV4G/LTR_p5_nrpe1.png]

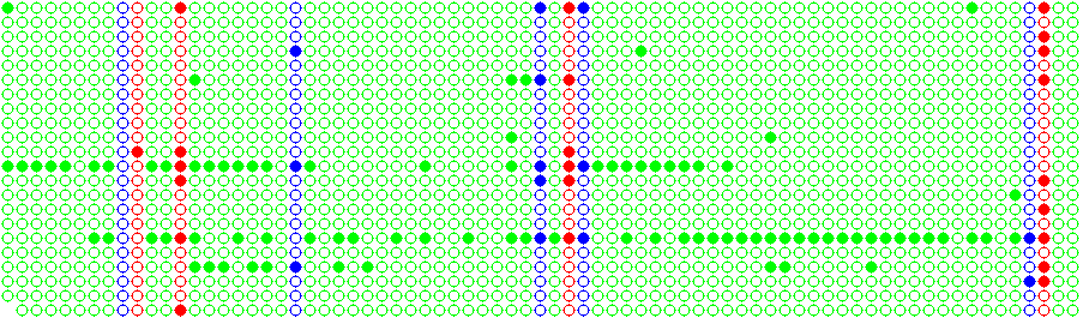

Supplement: Supplementary file 9 — Figure EV1-4 Source Data [file 44319_2024_304_MOESM9_ESM.zip › EV Figures/Figure EV4/EV4F/LTR_p4_F6-.png]

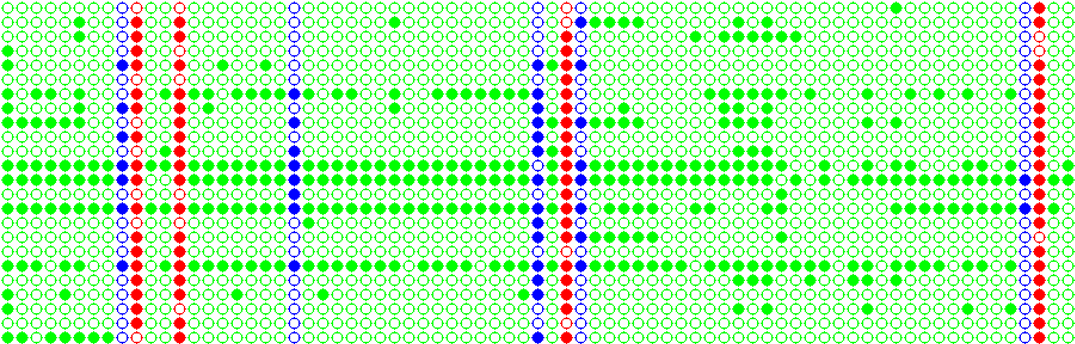

Supplement: Supplementary file 9 — Figure EV1-4 Source Data [file 44319_2024_304_MOESM9_ESM.zip › EV Figures/Figure EV4/EV4F/LTR_p4_F6+.png]

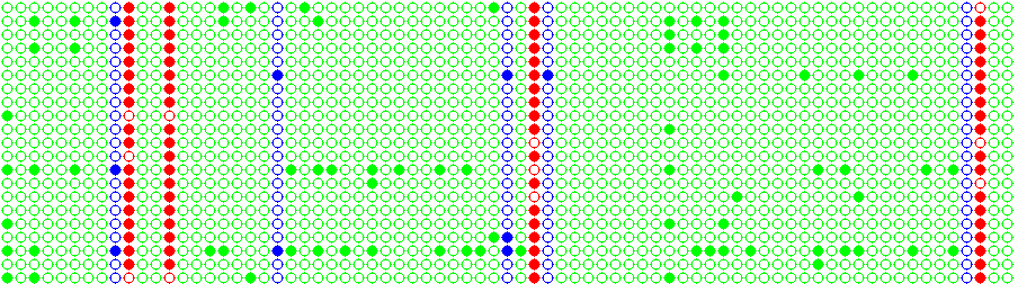

Supplement: Supplementary file 9 — Figure EV1-4 Source Data [file 44319_2024_304_MOESM9_ESM.zip › EV Figures/Figure EV4/EV4F/LTR_p4_nrpd1.png]

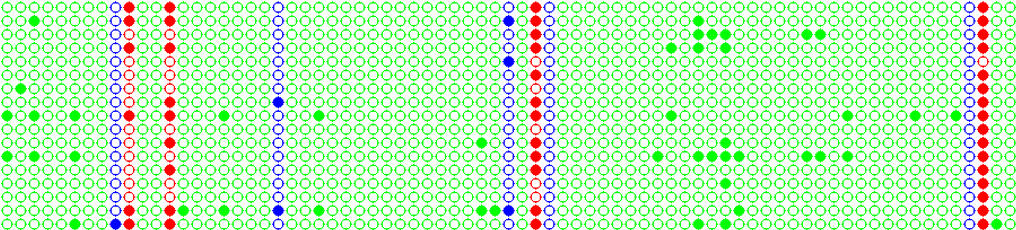

Supplement: Supplementary file 9 — Figure EV1-4 Source Data [file 44319_2024_304_MOESM9_ESM.zip › EV Figures/Figure EV4/EV4F/LTR_p4_Col-0.png]

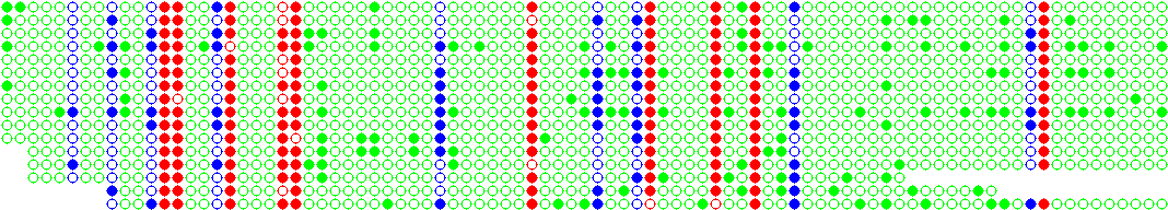

Supplement: Supplementary file 9 — Figure EV1-4 Source Data [file 44319_2024_304_MOESM9_ESM.zip › EV Figures/Figure EV4/EV4D/GAG_p4_Col-0.png]

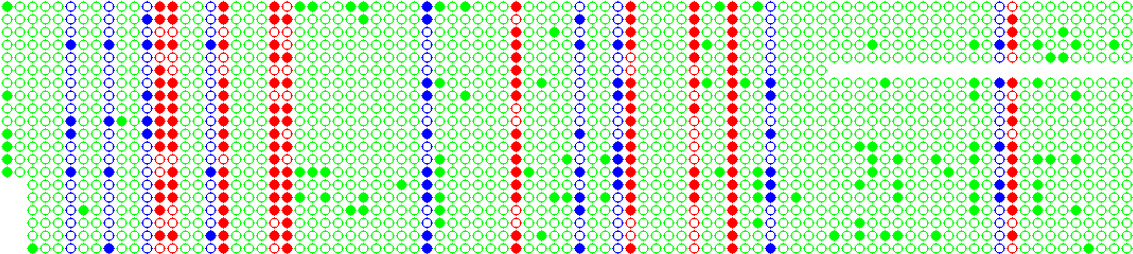

Supplement: Supplementary file 9 — Figure EV1-4 Source Data [file 44319_2024_304_MOESM9_ESM.zip › EV Figures/Figure EV4/EV4D/GAG_p4_F6+.png]

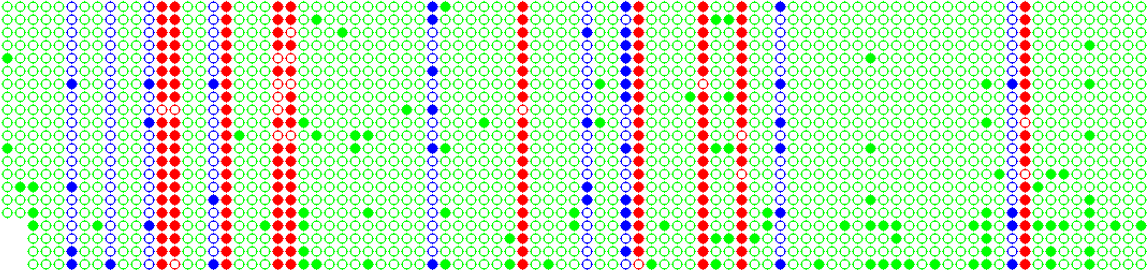

Supplement: Supplementary file 9 — Figure EV1-4 Source Data [file 44319_2024_304_MOESM9_ESM.zip › EV Figures/Figure EV4/EV4D/GAG_p4_nrpd1.png]

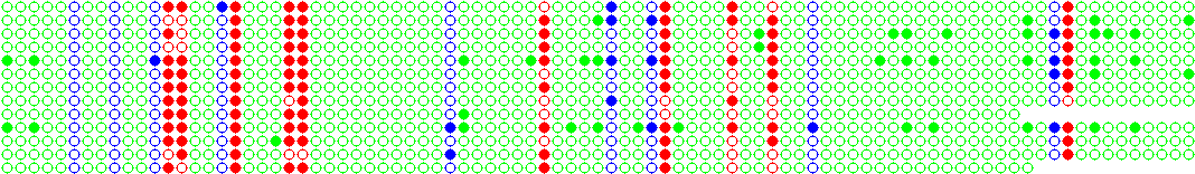

Supplement: Supplementary file 9 — Figure EV1-4 Source Data [file 44319_2024_304_MOESM9_ESM.zip › EV Figures/Figure EV4/EV4D/GAG_p4_F6-.png]

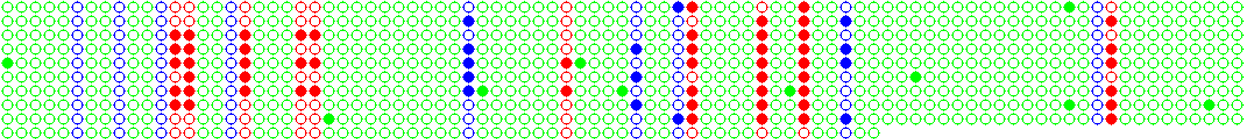

Supplement: Supplementary file 9 — Figure EV1-4 Source Data [file 44319_2024_304_MOESM9_ESM.zip › EV Figures/Figure EV4/EV4E/GAG_p5_F6-.png]

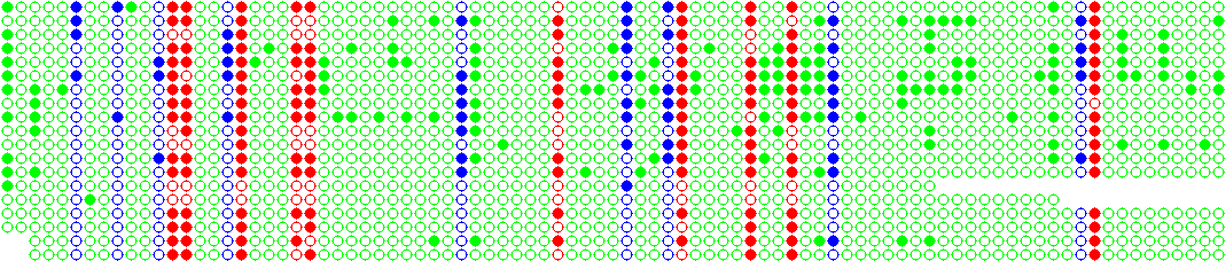

Supplement: Supplementary file 9 — Figure EV1-4 Source Data [file 44319_2024_304_MOESM9_ESM.zip › EV Figures/Figure EV4/EV4E/GAG_p5_F6+.png]

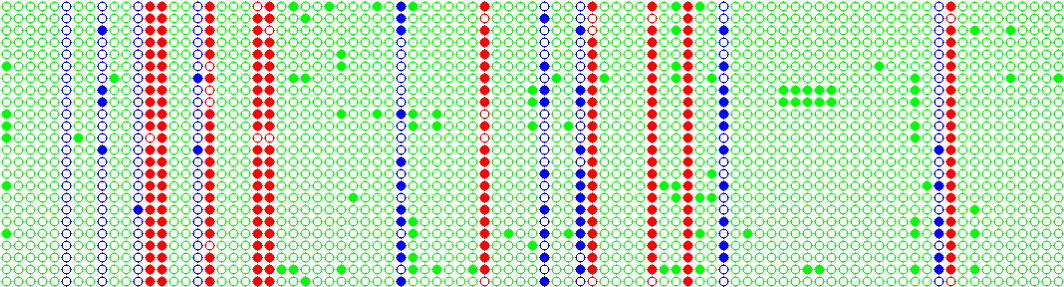

Supplement: Supplementary file 9 — Figure EV1-4 Source Data [file 44319_2024_304_MOESM9_ESM.zip › EV Figures/Figure EV4/EV4E/GAG_p5_nrpe1.png]

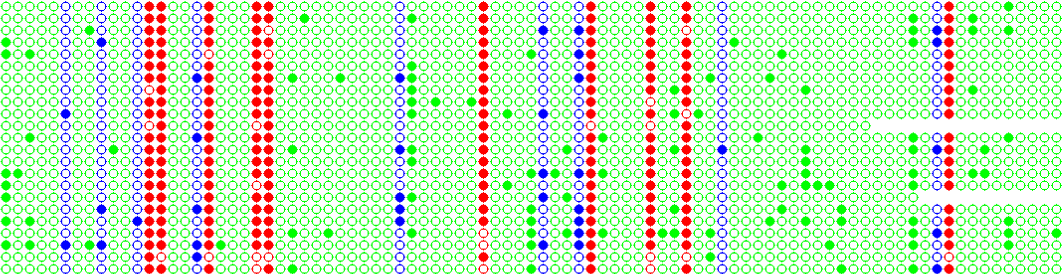

Supplement: Supplementary file 9 — Figure EV1-4 Source Data [file 44319_2024_304_MOESM9_ESM.zip › EV Figures/Figure EV4/EV4E/GAG_p5_Col-0.png]
