# Supplementary material for: PTGS is dispensable for the initiation of epigenetic silencing of an active transposon in Arabidopsis
Source: EMBO Rep. 2024 Nov 7;25(12):28. doi: 10.1038/s44319-024-00304-5 (PMC11624286; doi:10.1038/s44319-024-00304-5)
Supplement: Supplementary file 3 — Source data Fig. 2 [file 44319_2024_304_MOESM3_ESM.zip › Figure 2/2A/Northern blots 2A.pdf]

Same membranes were re-probed multiple times against indicated targets, cropped areas for inclusion in the final figure are indicated in red squares. (lane/sample description at the end of the document)

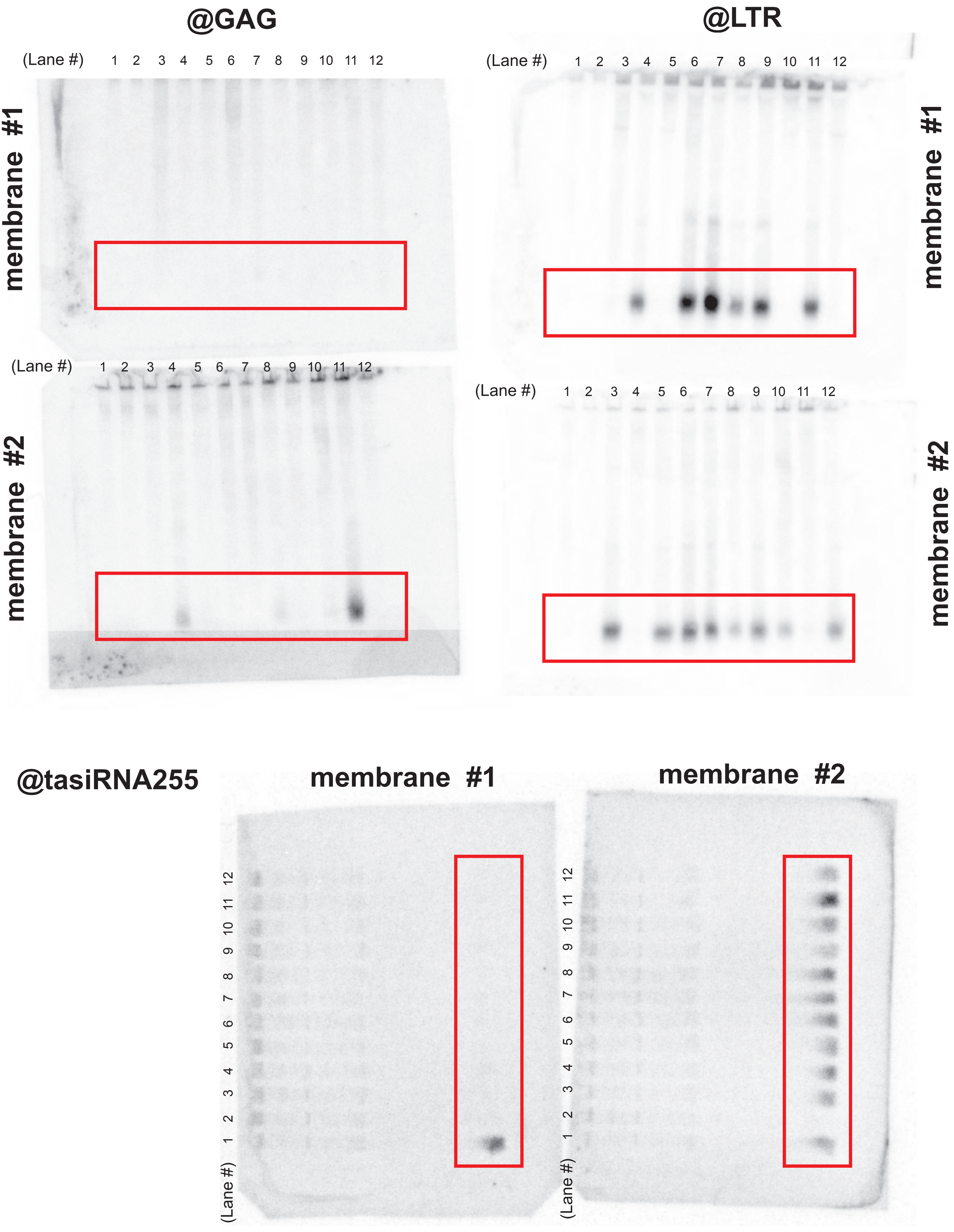

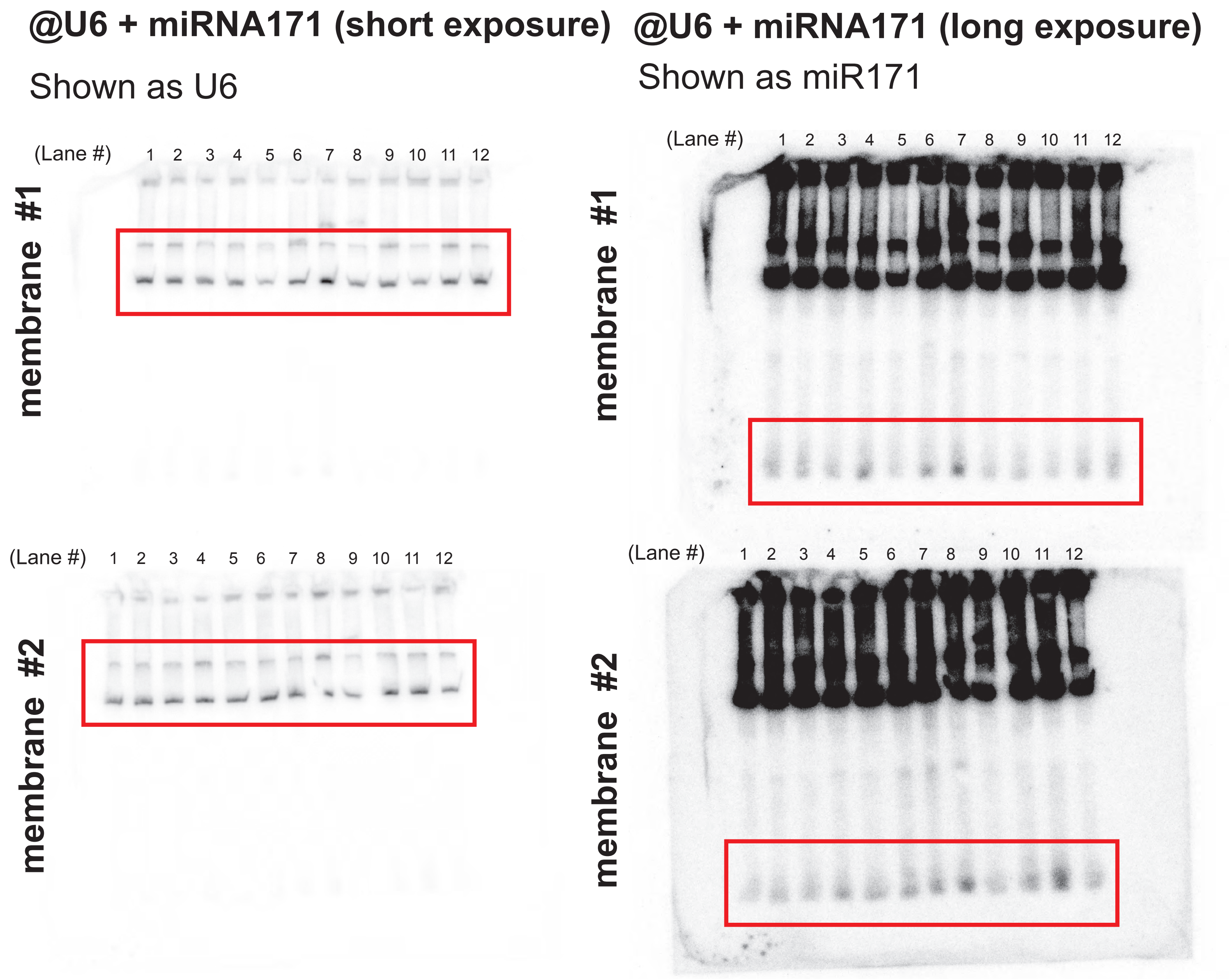

Lane sample loading:

rdr6 x epi15 *EVD-rdr6* (**membrane 1**) and *EVD-RDR6* (**membrane 2**) F6 individuals

MEMBRANE 1:

1) Col-0
2) *rdr6*
3) F6 *rdr6 indv.#1*
4) F6 *rdr6 indv.#2*
5) F6 *rdr6 indv.#3*
6) F6 *rdr6 indv.#4*
7) F6 *rdr6 indv.#5*
8) F6 *rdr6 indv.#6*
9) F6 *rdr6 indv.#7*
10) F6 *rdr6 indv.#8*
11) F6 *rdr6 indv.#9*
12) F6 *rdr6 indv.#10*

MEMBRANE 2:

1) Col-0
2) *rdr6*
3) F6 *RDR6 indv.#1*
4) F6 *RDR6 indv.#2*
5) F6 *RDR6 indv.#3*
6) F6 *RDR6 indv.#4*
7) F6 *RDR6 indv.#5*
8) F6 *RDR6 indv.#6*
9) F6 *RDR6 indv.#7*
10) F6 *RDR6 indv.#8*
11) F6 *RDR6 indv.#9*
12) F6 *RDR6 indv.#10*
