# Supplementary material for: PTGS is dispensable for the initiation of epigenetic silencing of an active transposon in Arabidopsis
Source: EMBO Rep. 2024 Nov 7;25(12):28. doi: 10.1038/s44319-024-00304-5 (PMC11624286; doi:10.1038/s44319-024-00304-5)
Supplement: Supplementary file 3 — Source data Fig. 2 [file 44319_2024_304_MOESM3_ESM.zip › Figure 2/2A/Raw blot images 2A/Fig2A_pictures_description.rtf]

Raw files for northerns of Figure 2 - ATop membrane -  F6 rdr6 (-/-) x epi15 F8 line, individual rdr6 mutant plants with active EVD copiesLoading (form left to right) - Col-0, rdr6, ind#1, ind#2, ind#3, ind#4, ind#5, ind#6, ind#7, ind#8, ind#9, ind#10Lower membrane -  F6 RDR6 (+/+) x epi15 F8 line, individual RDR6 wild-type plants with active EVD copiesLoading (form left to right) - Col-0, rdr6, ind#1, ind#2, ind#3, ind#4, ind#5, ind#6, ind#7, ind#8, ind#9, ind#10
