# Supplementary material for: PTGS is dispensable for the initiation of epigenetic silencing of an active transposon in Arabidopsis
Source: EMBO Rep. 2024 Nov 7;25(12):28. doi: 10.1038/s44319-024-00304-5 (PMC11624286; doi:10.1038/s44319-024-00304-5)
Supplement: Supplementary file 7 — Source data Fig. 6 [file 44319_2024_304_MOESM7_ESM.zip › Figure 6/6D/Northern blots 6D.pdf]

Same membrane was reprobed multiple times against indicated targets, cropped areas for inclusion in the final figure are indicated in red squares. (lane/sample description at the end of the document)

@GAG

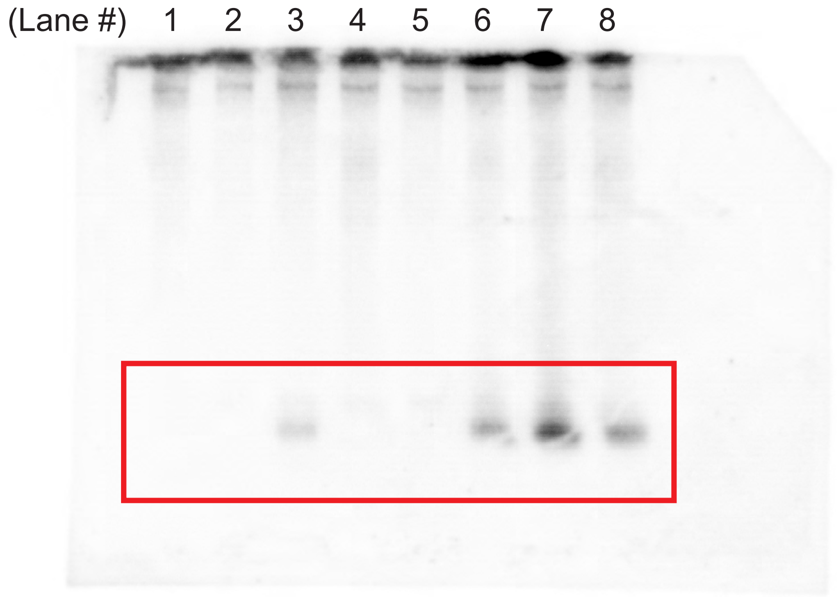

@LTR

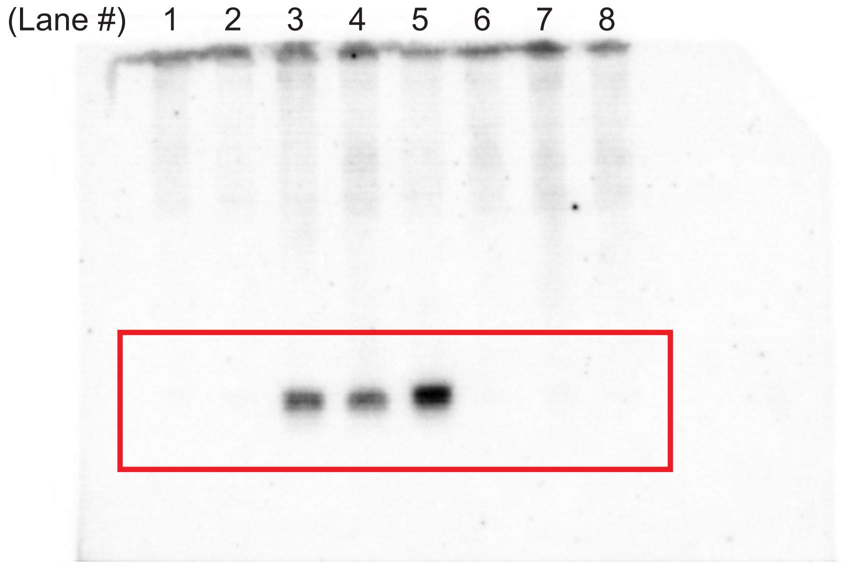

@siRNA1003

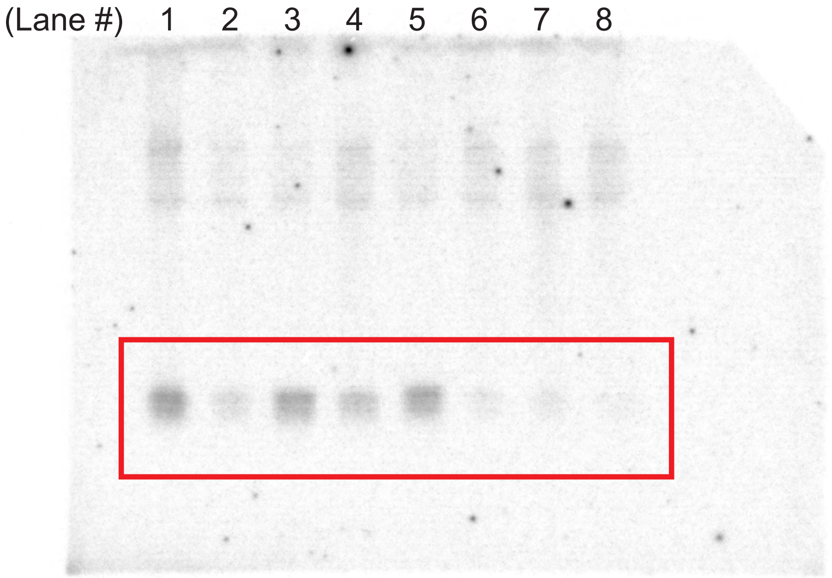

@U6 + miRNA171

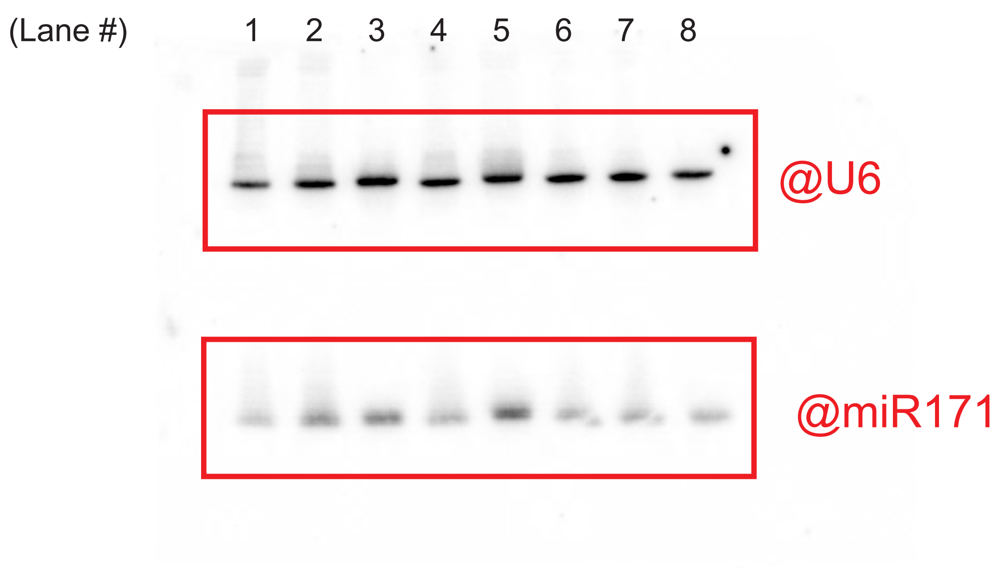

Lane sample loading:

nrpe1 x epi15 bulks of mutant and WT plants at generation F6

1) Col-0
2) *nrpe1*
3) *NRPE1-EVD* F6 bulk #1
4) *NRPE1-EVD* F6 bulk #2
5) *NRPE1-EVD* F6 bulk #3
6) *nrpe1-EVD* F6 bulk #1
7) *nrpe1-EVD* F6 bulk #2
8) *nrpe1-EVD* F6 bulk #3
