# Supplementary material for: PTGS is dispensable for the initiation of epigenetic silencing of an active transposon in Arabidopsis
Source: EMBO Rep. 2024 Nov 7;25(12):28. doi: 10.1038/s44319-024-00304-5 (PMC11624286; doi:10.1038/s44319-024-00304-5)
Supplement: Supplementary file 7 — Source data Fig. 6 [file 44319_2024_304_MOESM7_ESM.zip › Figure 6/6D/Raw blot images 6D/Info_northern_pictures_Fig5D.rtf]

Raw files for northerns of Figure 5 DMembrane 008 -  F6 pol5-EVD x epi15 F11 line, three replicates for Pol5 (+/+) and pol5 (-/-) lines with active EVD Loading (form left to right) - Col-0, nrpe1, pol5(+/+) rep1, pol5(+/+) rep2, pol5(+/+) rep3, pol5(-/-) rep1, pol5(-/-) rep2, pol5(-/-) rep3
