# Supplementary material for: PTGS is dispensable for the initiation of epigenetic silencing of an active transposon in Arabidopsis
Source: EMBO Rep. 2024 Nov 7;25(12):28. doi: 10.1038/s44319-024-00304-5 (PMC11624286; doi:10.1038/s44319-024-00304-5)
Supplement: Supplementary file 7 — Source data Fig. 6 [file 44319_2024_304_MOESM7_ESM.zip › Figure 6/6C/Raw blot images 6C/Info_northern_pictures_Fig5C.rtf]

Raw files for northerns of Figure 5 CMembrane MT02 -  F6 pol4-EVD x epi15 F11 line, three replicates for Pol4 (+/+) and pol4 (-/-) lines with active EVD Loading (form left to right) - Col-0, nrpd1, pol4(+/+) rep1, pol4(+/+) rep2, pol4(+/+) rep3, pol4(-/-) rep1, pol4(-/-) rep2, pol4(-/-) rep3
