# Supplementary material for: PTGS is dispensable for the initiation of epigenetic silencing of an active transposon in Arabidopsis
Source: EMBO Rep. 2024 Nov 7;25(12):28. doi: 10.1038/s44319-024-00304-5 (PMC11624286; doi:10.1038/s44319-024-00304-5)
Supplement: Supplementary file 10 — Expanded View Figures [file 44319_2024_304_MOESM10_ESM.pdf]

Expanded View Figures

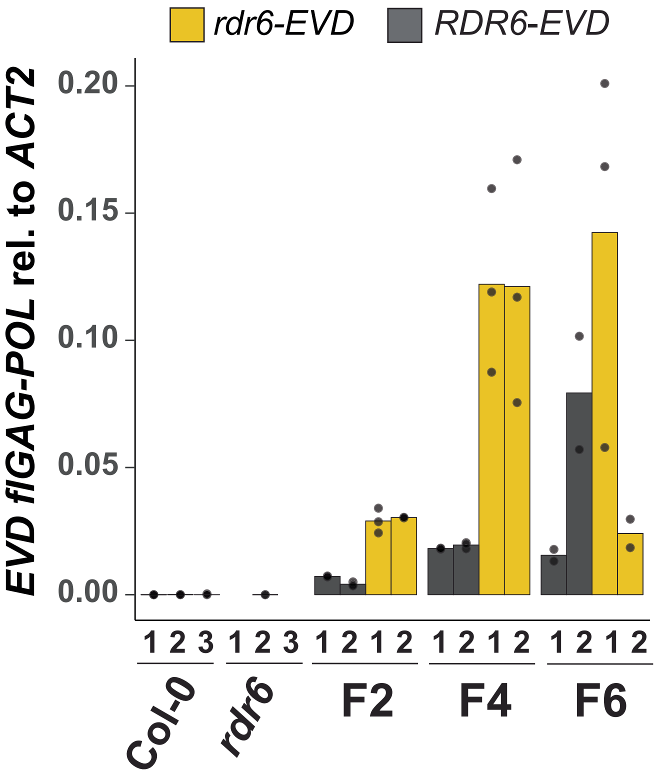

Figure EV1. ***EVD GAG-POL*** **expression in RDR6 wild-type and mutant backgrounds.**

(**A**) qPCR analysis of *flGAG-POL* expression normalized to *ACT2* in *EVD*-RDR6 and *EVD-rdr6* lines at generations 2, 4, and 6 derived from two independent F1s (biological replicates). Each biological replicate, consistent of bulks of 8–10 plants, are represented for each genotype at each generation, dots show technical replicates.

© The Author(s)

A

sample	conversion rate
Col-0 #1	99,857 %
Col-0 #2	99,866 %
rdr6-15 #1	99,847 %
rdr6-15 #2	99,861 %
RDR6-EVD #2	99,850 %
RDR6-EVD #9	99,870 %
RDR6-EVD #1	99,878 %
RDR6-EVD #7	99,862 %
rdr6-EVD #8	99,864 %
rdr6-EVD #10	99,830 %
rdr6-EVD #5	99,880 %
rdr6-EVD #6	99,865 %

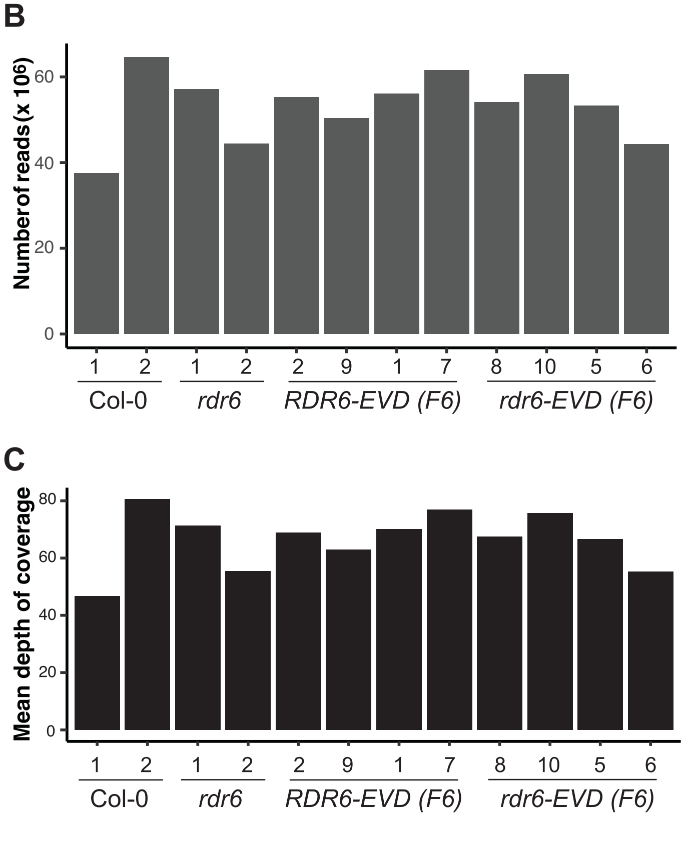

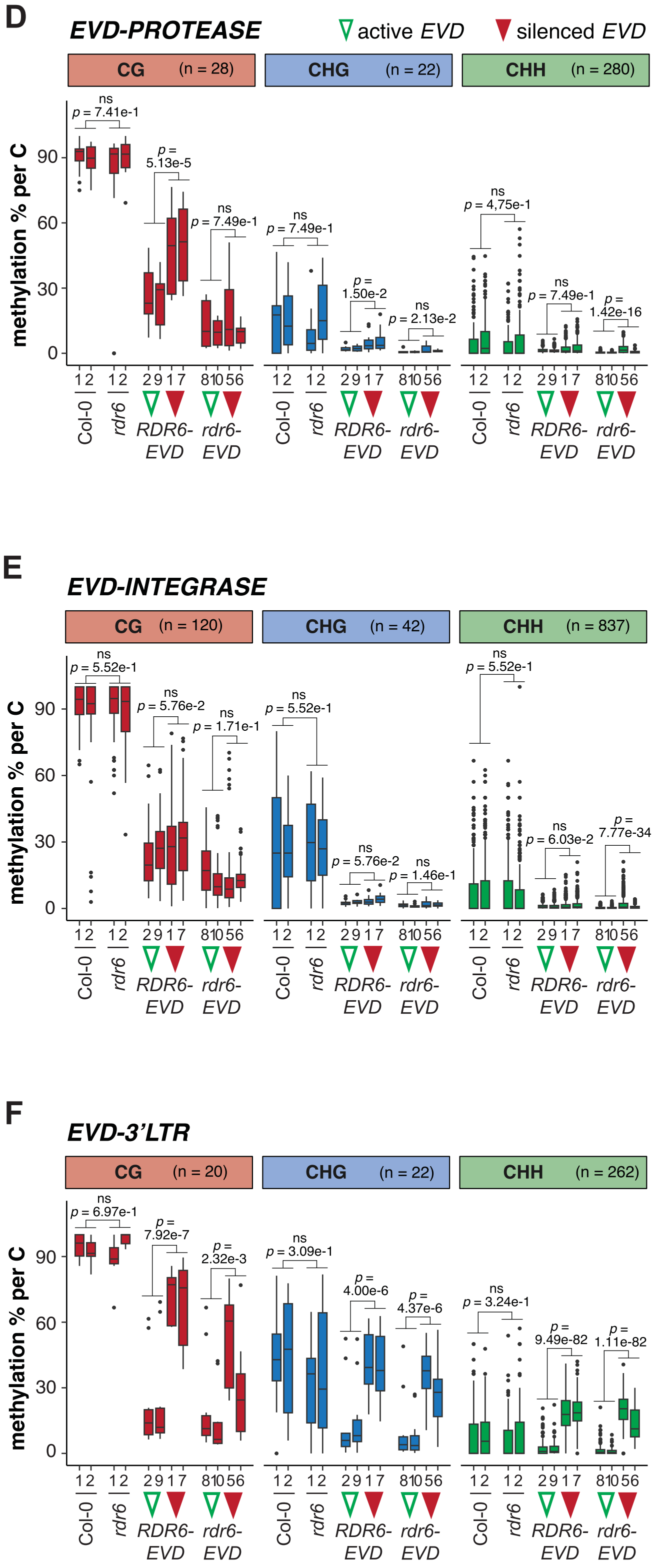

 © The Author(s)

Figure EV2. EM-seq libraries general stats and *EVD POL* and *3′LTR* methylation in *RDR6*- and *rdr6-EVD* lines.

(**A**) Cytosine-to-thymine conversion rates of the unmethylated chloroplastic DNA for each EM-seq library (see materials and methods for further information). (**B**) Number of paired-end fragments obtained in each EM-seq library. (**C**) *Arabidopsis* genome coverage in each EM-seq library. (**D–F**) EM-seq analysis of DNA methylation (as % per of methylated cytosines) in CG, CHG, and CHH contexts in WT Col-0, *rdr6* and in *RDR6*- and *rdr6-EVD* F6 individuals with active and silenced *EVD* (marked with empty green and filled red arrowheads, respectively, numbers indicate same individuals as in Fig. 2) in: (**D**) *EVD-Protease*; (**E**) *EVD-Integrase,* and (**F**) *EVD-5′LTR*. In panels (**D–F**), *n* indicates the number of cytosines analyzed for each context per sample. In all boxplots: median is indicated by a solid bar, the boxes extend from the first to the third quartile and whiskers reach to the furthest values within 1.5 times the interquartile range. Dots indicate outliers, as data points outside of the above range. Wilcoxon rank sum test adjusted *p*-values between indicated groups of samples are shown. Differences are considered statistically significant if $p < 0.05$ (5.00e−2) or non-significant (ns) if $p \geq 0.05$.

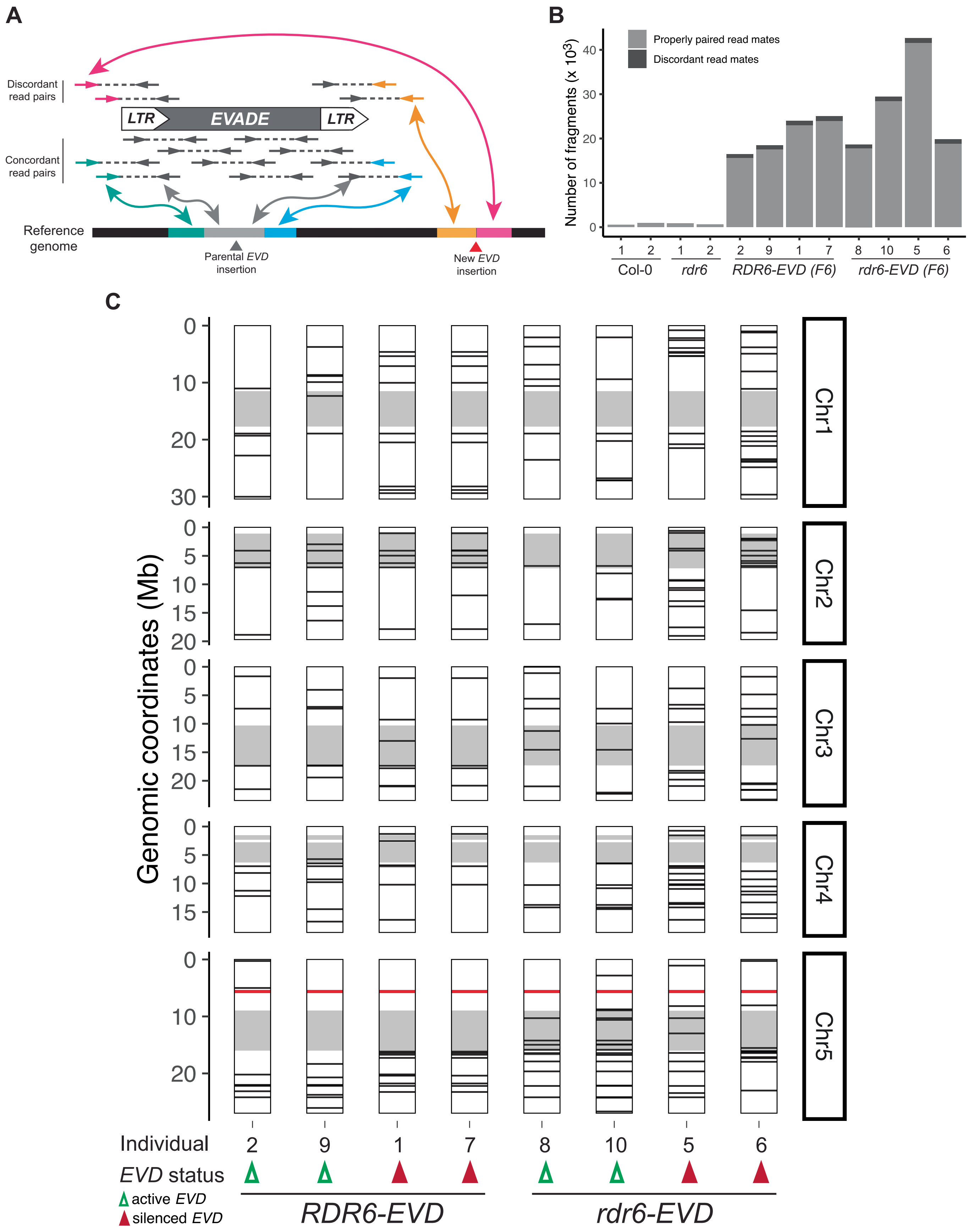
A
Discordant read pairs
LTR
EVADE
LTR
Concordant read pairs
Reference genome
Parental EVD insertion
New EVD insertion
B
Properly paired read mates
Discordant read mates
Number of fragments (x 10³)
40
30
20
10
0
1 2 1 2 2 9 1 7 8 10 5 6
Col-0
rdr6
RDR6-EVD (F6)
rdr6-EVD (F6)
C
Genomic coordinates (Mb)
Chr1
Chr2
Chr3
Chr4
Chr5
Individual
2 9 1 7 8 10 5 6
EVD status
active EVD
silenced EVD
RDR6-EVD
rdr6-EVD

 © The Author(s)

◀ **Figure EV3. Mapping of new *EVD* insertions in *RDR6*- and *rdr6-EVD* lines from EM-seq data.**

(**A**) Schematic representation of the strategy used to map new *EVD* insertions using discordant read mates from EM-seq. (**B**) Number of fragments from concordant (properly paired) and discordant read mates mapping to EVD in each of the EM-seq libraries. (**C**) Genomic location of new EVD insertions mapped through discordant read pair mates in EM-seq data in the *Arabidopsis* genome. Parental EVD (AT5G17125) location is indicated with a red line. New EVD insertions are marked with black lines. Pericentromeric regions in each of *Arabidopsis* five chromosomes are marked in gray. See table provided in corresponding source data for precise chromosome coordinates of each new insertion.

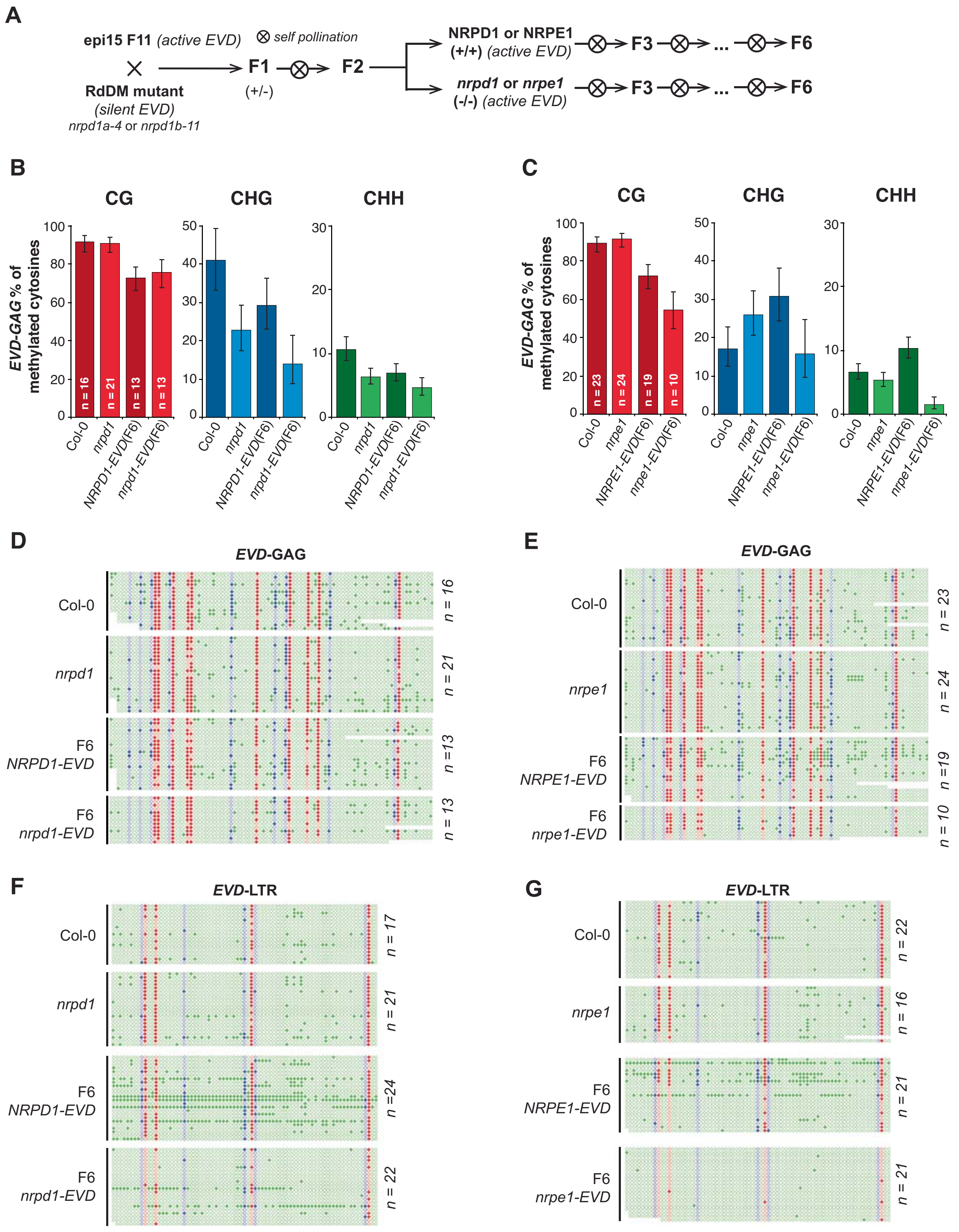
A
epi15 F11 (active EVD)
RdDM mutant
(silent EVD)
nrpd1a-4 or nrpd1b-11
⊗ self pollination
F1
(+/-)
F2
NRPD1 or NRPE1
(+/+) (active EVD)
nrpd1 or nrpe1
(-/-) (active EVD)
F3
F6
B
C
CG
CHG
CHH
EVD-GAG % of methylated cytosines
Col-0
nrpd1
NRPD1-EVD(F6)
nrpd1-EVD(F6)
nrpe1
NRPE1-EVD(F6)
nrpe1-EVD(F6)
n = 16
n = 21
n = 13
n = 23
n = 24
n = 19
n = 10
D
E
EVD-GAG
F6
NRPD1-EVD
nrpd1-EVD
NRPE1-EVD
nrpe1-EVD
F
G
EVD-LTR
n = 17
n = 24
n = 22
n = 16
n = 21

 © The Author(s)

Figure EV4. BS-PCR analysis of EVD-GAG DNA methylation levels in RdDM mutants.

(**A**) Crossing scheme to generate nrpd1- and nrpe1-EVD lines. F2 plants were genotyped to select homozygous WT and mutant lines for each background and propagated through selfing until the F6 generation. (**B**, **C**) % methylated cytosines by bisulfite -PCR DNA methylation analysis at EVD-GAG sequences in the F6 generation of *NRPD1-EVD* (**B**) and *NRPE1-EVD* (**C**) lines, in both WT (darker shade) and mutant (lighter shade) backgrounds. Col-0, *nrpd1* and *nrpe1* were used as controls. Error bars represent 95% confidence Wilson score intervals of the % of methylated cytosines (C) in each context (CG, CHG, CHH). (**D**, **E**) Dot-plot representation of bisulfite-PCR sequencing data for the F6 generation of *NRPD1-EVD* (**D**) and *NRPE1-EVD* (**E**) lines, in both WT and mutant backgrounds, at *EVD-GAG* sequences. Col-0 and *nrpd1* or *nrpe1* were used as control for the endogenous parental *EVD* copies. (**F**, **G**) Dot-plot representation of bisulfite-PCR sequencing data for the F6 generation of *NRPD1-EVD* (**F**) and *NRPE1-EVD* (**G**) lines, in both WT and mutant backgrounds, at *EVD-LTR* sequences. Col-0 and *nrpd1* or *nrpe1* were used as control for the endogenous parental *EVD* copies. In all dot-plots, filled circle represent methylated, empty circles unmethylated cytosines in the CG (red), CHG (blue), and CHH (green) context.

INSERTION ORIGIN		STRUCTURE	HAIRPIN	OCCURENCE							
				RDR6-EVD (F6)				*rdr6-EVD* (F6)			
Tandem	Nested			2	9	1	7	8	10	5	6
Tail-to-Tail 3'-to-3' *LTR*	in Antisense 5'-to-3' *LTR*	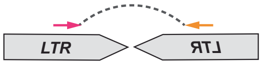	YES	N	N	N	N	N	N	N	N
Head-to-Head 5'-to-5' *LTR*	in Antisense 5'-to-3' *LTR*	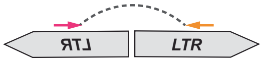	YES	N	N	N	N	N	N	N	N
Head-to-Tail 3'-to-5' *LTR*	in Sense 5'-to-5' *LTR* 3'-to-3' *LTR*	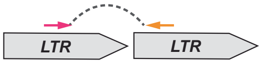	NO	N	N	N	N	N	N	N	N

Figure EV5. Summary table for the search of close proximity sense-antisense *EVD-LTR* events.

Putative origin of *LTR* hairpins as consequence of *EVD* transposition in tandem or nested configurations next to the scheme of the strategy used to find them using discordant read mates from EM-seq where both read mates map to *EVD-LTR*. The table indicates the occurrence and number of read mates found in each sample. Arbitrary threshold of at least three paired read mates was set to confidentially identify two *LTR*s in close proximity. N: non-occurrence, y: presence of at least one discordant paired read mate, Y: presence of discordant paired read mates and above threshold for confident calling.

© The Author(s)
